# Oncolytic virus-mediated p53 overexpression promotes immunogenic cell death and efficacy of PD-1 blockade in pancreatic cancer

**DOI:** 10.1016/j.omto.2022.09.003

**Published:** 2022-09-13

**Authors:** Hiroyuki Araki, Hiroshi Tazawa, Nobuhiko Kanaya, Yoshinori Kajiwara, Motohiko Yamada, Masashi Hashimoto, Satoru Kikuchi, Shinji Kuroda, Ryuichi Yoshida, Yuzo Umeda, Yasuo Urata, Shunsuke Kagawa, Toshiyoshi Fujiwara

**Affiliations:** 1 Department of Gastroenterological Surgery, Okayama University Graduate School of Medicine, Dentistry and Pharmaceutical Sciences, Okayama, Japan; 2 Center for Innovative Clinical Medicine, Okayama University Hospital, Okayama, Japan; 3 Minimally Invasive Therapy Center, Okayama University Hospital, Okayama, Japan; 4 Oncolys BioPharma, Inc., Tokyo, Japan

**Keywords:** pancreatic cancer, oncolytic adenovirus, p53, immunogenic cell death, PD-1 blockade

## Abstract

Immune checkpoint inhibitors, including anti-programmed cell death 1 (PD-1) antibody, provide improved clinical outcome in certain cancers. However, pancreatic ductal adenocarcinoma (PDAC) is refractory to PD-1 blockade therapy due to poor immune response. Oncolytic virotherapy is a novel approach for inducing immunogenic cell death (ICD). We demonstrated the therapeutic potential of p53-expressing telomerase-specific oncolytic adenovirus OBP-702 to induce ICD and anti-tumor immune responses in human PDAC cells with different p53 status (Capan-2, PK-59, PK-45H, Capan-1, MIA PaCa-2, BxPC-3) and murine PDAC cells (PAN02). OBP-702 significantly enhanced ICD with secretion of extracellular adenosine triphosphate and high-mobility group box protein B1 by inducing p53-mediated apoptosis and autophagy. OBP-702 significantly promoted the tumor infiltration of CD8+ T cells and the anti-tumor efficacy of PD-1 blockade in a subcutaneous PAN02 syngeneic tumor model. Our results suggest that oncolytic adenovirus-mediated p53 overexpression augments ICD and the efficacy of PD-1 blockade therapy against cold PDAC tumors. Further *in vivo* experiments would be warranted to evaluate the survival benefit of tumor-bearing mice in combination therapy with OBP-702 and PD-1 blockade.

## Graphical abstract

**Figure:**
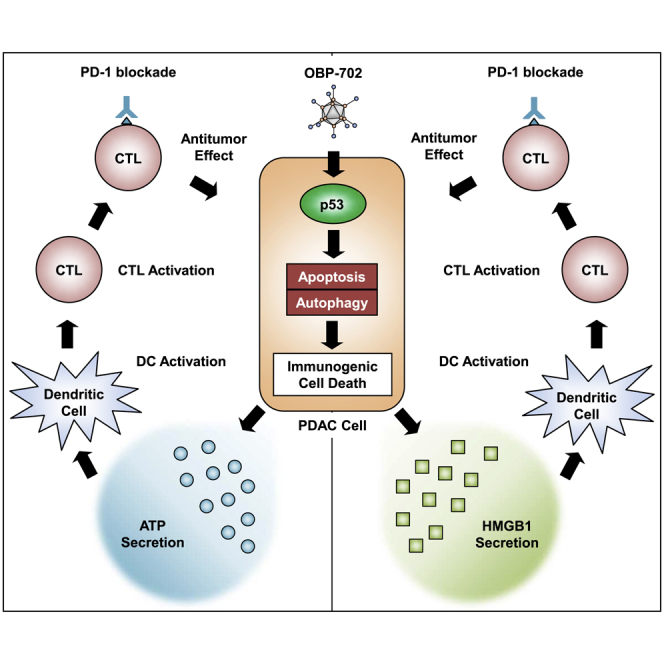

## Teaser

Pancreatic ductal adenocarcinoma (PDAC) is refractory to PD-1 blockade therapy due to poor immune response. Telomerase-specific oncolytic adenovirus OBP-702 induced profound immunogenic cell death with secretion of extracellular ATP and HMGB1 via p53-mediated induction of apoptosis and autophagy. OBP-702 enhanced the anti-tumor efficacy of PD-1 blockade therapy against PDAC tumors via induction of tumor-infiltrating CD8+ T cells.

## Introduction

Pancreatic ductal adenocarcinoma (PDAC) is one of the most lethal cancers. Despite recent advances in surgery, chemotherapy, radiotherapy, and molecular-targeted therapy, however, the overall 5-year survival rate is still less than 10%.^1^ Immunotherapy with immune checkpoint inhibitors (ICIs), including anti-programmed cell death-1 (PD-1)/programmed cell death ligand-1 (PD-L1) antibodies and anti-cytotoxic T lymphocyte-associated protein 4 (CTLA-4), were recently demonstrated to improve the clinical outcome of certain cancers.^2^ However, PDAC is less sensitive to immunotherapy due to low mutation burden and poor infiltration of cytotoxic T cells.^3^ Moreover, PDAC cells evade the anti-tumor immune system via massive stroma, poor angiogenesis, and infiltration of immunosuppressive cells.^3^ Therefore, novel therapeutic approaches to stimulate the anti-tumor immune response are needed to improve the therapeutic efficacy of immunotherapy in PDAC.

Immunogenic cell death (ICD) induces the release of damage-associated molecular patterns (DAMPs), including adenosine triphosphate (ATP) and high-mobility group box 1 (HMGB1), from dying cells. The induction of ICD stimulates the anti-tumor immune response, which enhances the anti-tumor efficacy of immunotherapy.^4^ ICD can be induced in response to various anti-tumor treatments, including chemotherapy, radiotherapy, and oncolytic virotherapy.^5^ Oncolytic virotherapy has recently emerged as a novel therapeutic approach for inducing profound ICD in combination with ICIs.^6^ We generated a telomerase-specific replication-competent oncolytic adenovirus, OBP-301 (Suratadenoturev), in which the human telomerase reverse transcriptase (*hTERT*) gene promoter drives expression of the *E1A* and *E1B* genes.^7^^,^^8^ OBP-301 induces tumor-specific lysis in association with autophagy in human cancer cells.^9^ Oncolytic adenoviruses have been shown to induce autophagy-related cell death in human cancer cells via modulation of E1- and E4-related signaling pathways.^10^ Recently, we demonstrated that an RGD fiber-modified OBP-301 variant (OBP-502) improves the anti-tumor efficacy of anti-PD-1 antibody in a syngeneic mouse model of colorectal and pancreatic cancer via induction of ICD and enhancement of CD8+ T cell tumor infiltration.^11^^,^^12^

Activation of the tumor suppressor p53 has been shown to induce ICD in p53-intact cancer cells after treatment with the MDM2 inhibitor Nutlin-3a.^13^ As p53 is frequently functionally inactivated in PDAC cells due to genetic alterations, restoration of p53 function may be an effective approach to induce ICD in PDAC cells. Adenovirus-mediated p53 gene therapy was developed to induce the expression of exogenous p53 protein in a variety of tumor cells.^14^ Kunimura et al. recently demonstrated that the replication-deficient p53-expressing adenovirus Ad-p53 enhances the anti-tumor efficacy of anti-PD-1 antibody in a syngeneic mouse model of urogenital cancer.^15^ We also generated a p53-expressing, telomerase-specific, replication-competent oncolytic adenovirus (OBP-702) in which the wild-type *p53* gene expression cassette was inserted into the E3 region of OBP-301.^16^^,^^17^ OBP-702 exhibits a profound anti-tumor effect in p53-mutant human PDAC cells by inducing p53-mediated apoptotic and autophagic cell death.^18^ Therefore, we hypothesized that OBP-702-mediated p53 overexpression would enhance the anti-tumor efficacy of anti-PD-1 antibody against PDAC cells via strong induction of ICD.

In the present study, we investigated the therapeutic potential of two different telomerase-specific oncolytic adenoviruses, OBP-301 and p53-armed OBP-702, and p53-expressing non-replicative adenovirus Ad-p53 (Figure S1) against p53-intact human PDAC cells (Capan-2, PK-59, PK-45H), p53-mutant human PDAC cells (Capan-1, MIA PaCa-2, BxPC-3), and murine PDAC cells (PAN02). Levels of extracellular ATP and HMGB1 were analyzed to evaluate virus-induced ICD. Virus-induced cytopathic activity was analyzed using XTT assays, and western blotting was used to analyze apoptosis and autophagy. A subcutaneous PAN02 syngeneic tumor model was used to evaluate virus-mediated immunomodulatory activity and the anti-tumor effect of combination therapy with OBP-702 and anti-PD-1 antibody.

## Results

### OBP-702 induces more profound ICD than OBP-301 in human PDAC cells with different p53 status

**Figure 1:**
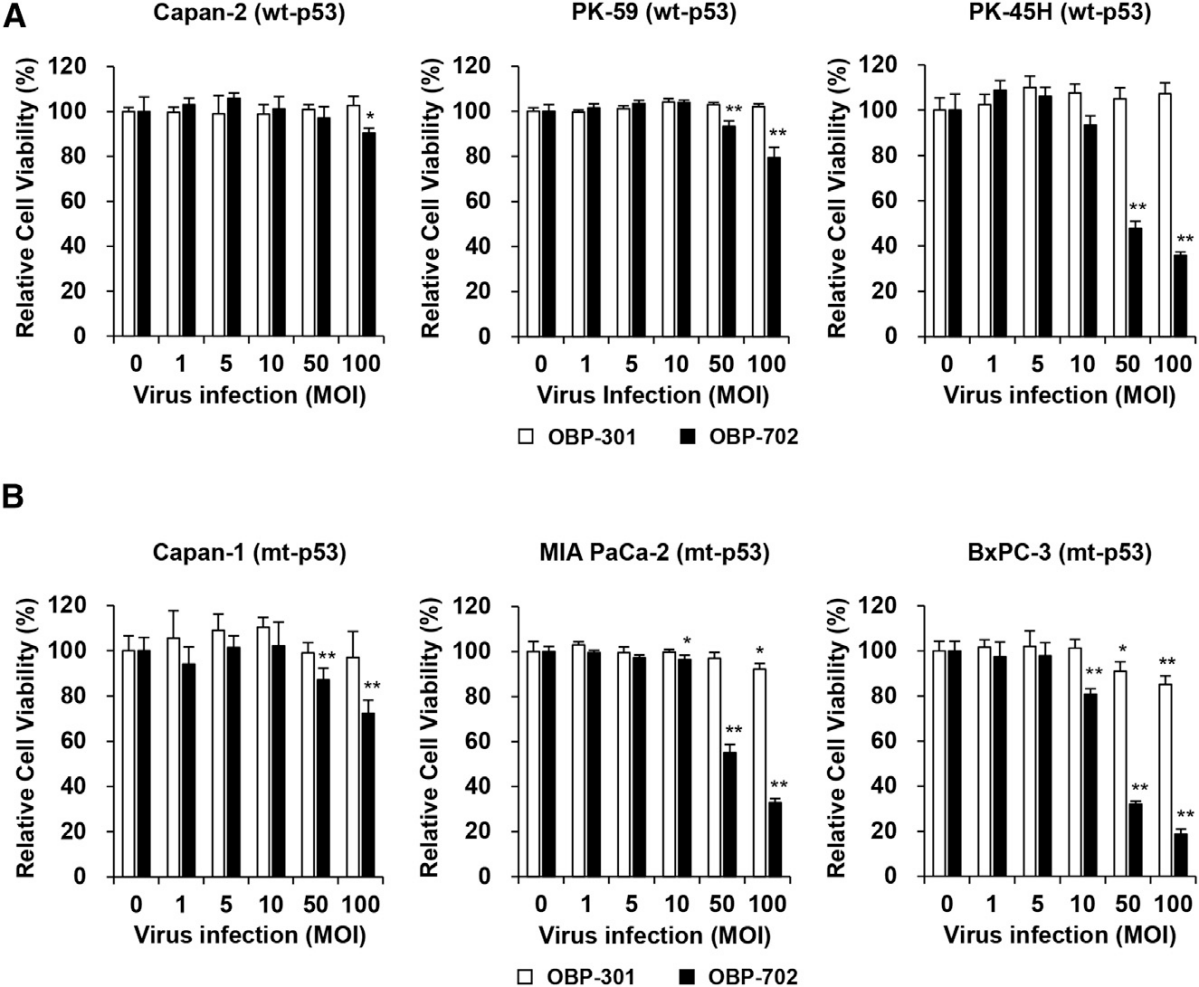
OBP-702 reduces the viability of human PDAC cells (A and B) Human PDAC cells with wild-type p53 (WT-p53) (A) and mutant p53 (mt-p53) (B) were infected with OBP-301 or OBP-702 at the indicated multiplicity of infection (MOI) for 48 h. Cell viability was quantified using the XTT assay. Uninfected (mock-treated) cells were shown as virus-infected cells at an MOI of 0. Cell viability was calculated relative to that of the mock-infected group, which was set at 100%. Cell viability data are expressed as mean ± SD (n = 5). Student’s t test was used to evaluate the significance of differences. ∗p < 0.05; ∗∗p < 0.01 (versus an MOI of 0).

To investigate the therapeutic potential of oncolytic adenoviruses against human PDAC cells, we used three p53-intact human PDAC cell lines (Capan-2, PK-59, PK-45H) and three p53-mutant human PDAC cell lines (Capan-1, MIA PaCa-2, BxPC-3). The viability of PDAC cells after infection with OBP-301 or OBP-702 for 48 and 120 h was assessed using an XTT assay. Infection with OBP-301 at high doses (multiplicity of infection [MOI] of 50 and 100) significantly suppressed the viability of MIA PaCa-2 and BxPC-3 cells 48 h after infection, whereas the viability of human PDAC cells, except for PK-59 cells, was significantly decreased 120 h after infection (Figures 1 and S2). OBP-702 significantly suppressed the viability of all human PDAC cells more strongly than OBP-301 48 and 120 h after infection (Figures 1 and S2). The OBP-702-mediated cytopathic effect was highly variable within p53-intact and p53-mutant human PDAC cells. Among human PDAC cells with different p53 status, p53-mutant BxPC-3 cells were most sensitive to the virus-mediated cytopathic effect.

**Figure 2:**
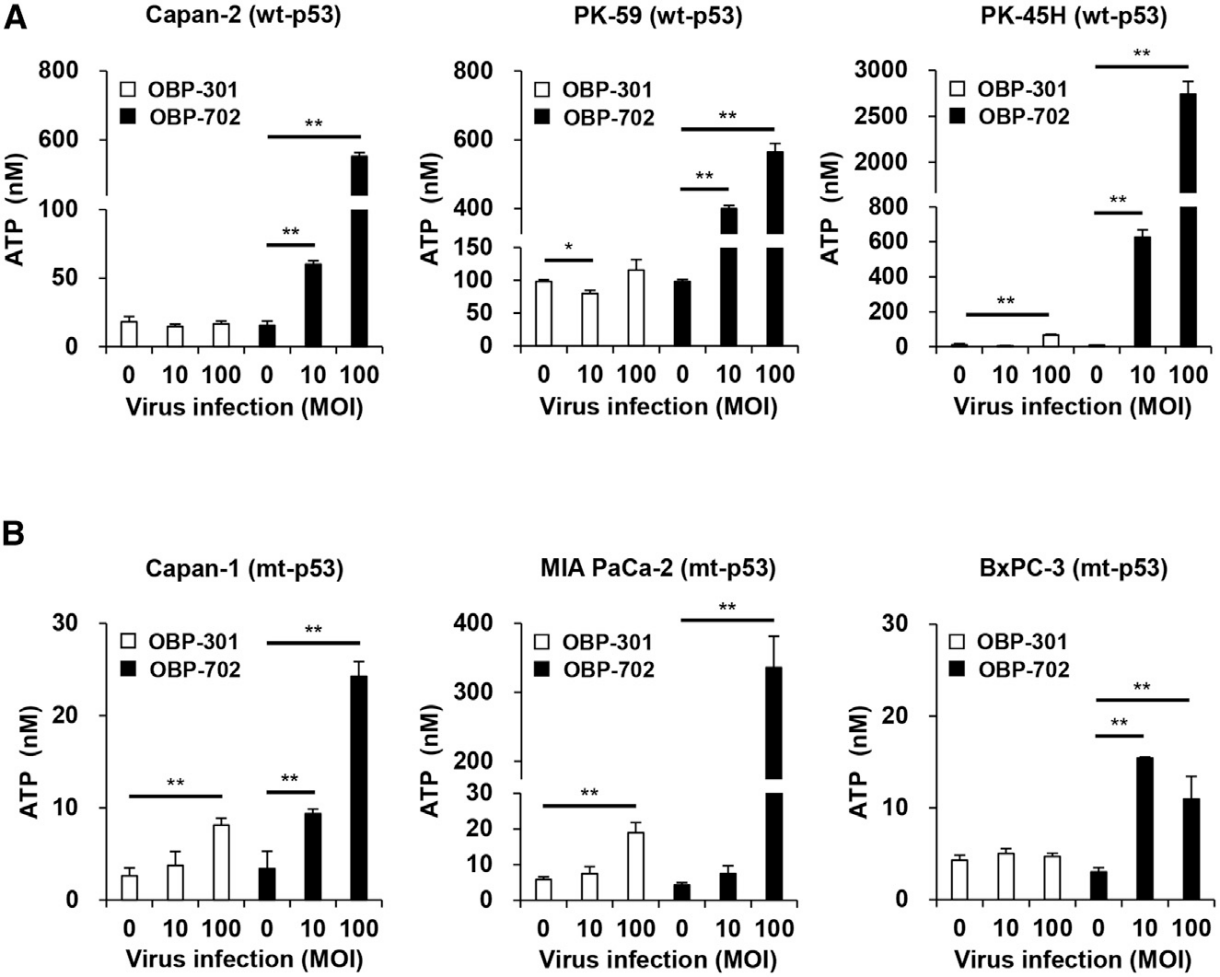
OBP-702 induces immunogenic cell death with ATP release in human PDAC cells (A and B) Human PDAC cells with WT-p53 (A) and mt-p53 (B) were treated with OBP-301 or OBP-702 at the indicated MOI for 48 h. Supernatants were collected, and the level of extracellular ATP was determined using an ENLITEN ATP assay. Uninfected (mock-treated) cells were shown as virus-infected cells at an MOI of 0. Data are expressed as mean ± SD (n = 3). Student’s t test was used to evaluate the significance of differences. ∗p < 0.05; ∗∗p < 0.01 (versus an MOI of 0).

**Figure 3:**
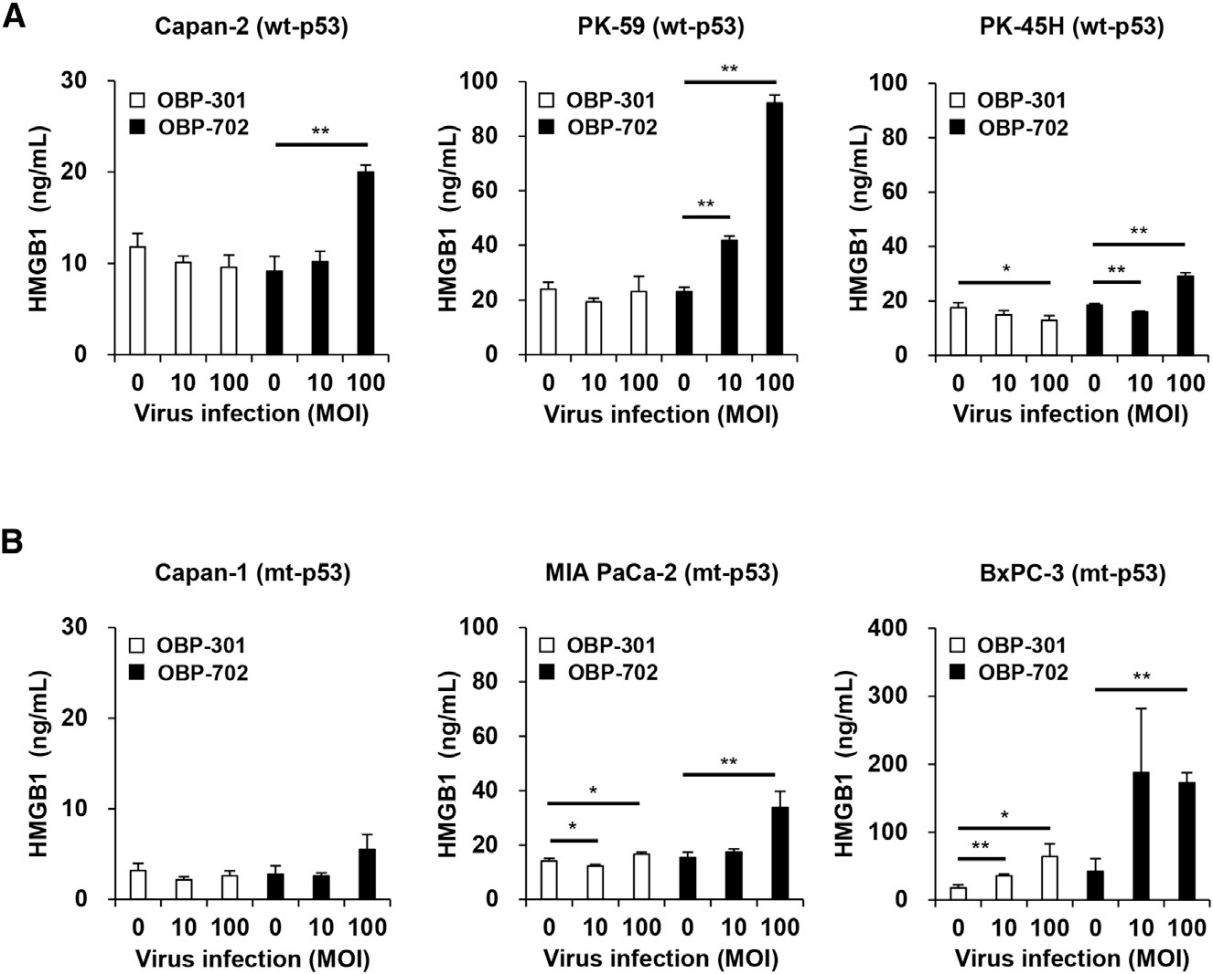
OBP-702 induces immunogenic cell death with HMGB1 release in human PDAC cells (A and B) Human PDAC cells with WT-p53 (A) and mt-p53 (B) were treated with OBP-301 or OBP-702 at the indicated MOI for 48 h. Supernatants were collected, and the level of extracellular HMGB1 was determined using an HMGB1 ELISA. Uninfected (mock-treated) cells were shown as virus-infected cells at an MOI of 0. Data are expressed as mean ± SD (n = 3). Student’s t test was used to evaluate the significance of differences. ∗p < 0.05; ∗∗p < 0.01 (versus an MOI of 0).

To evaluate the ICD-inducing activity of oncolytic adenoviruses against human PDAC cells, the levels of extracellular ATP and HMGB1 were determined using conditioned medium from human PDAC cells after infection with OBP-301 or OBP-702 for 48 h. OBP-301 significantly increased the level of extracellular ATP in p53-intact PK-45H cells and p53-mutant Capan-1 and MIA PaCa-2 cells (Figures 2A and 2B). OBP-702 significantly increased the release of extracellular ATP in p53-intact and p53-mutant human PDAC cells compared with OBP-301 (Figures 2A and 2B). The level of extracellular ATP released by p53-intact human PDAC cells was much higher than that released by p53-mutant PDAC cells (Figures 2A and 2B). In contrast, OBP-301 significantly increased the level of extracellular HMGB1 in p53-mutant MIA PaCa-2 and BxPC-3 cells (Figures 3A and 3B). OBP-702 induced significantly greater release of extracellular HMGB1 by p53-intact and p53-mutant human PDAC cells compared with OBP-301 (Figures 3A and 3B). Among human PDAC cells with different p53 status, p53-mutant BxPC-3 cells released the highest levels of HMGB1 (Figures 3A and 3B). To further evaluate the ICD-inducing activity of oncolytic adenoviruses, the proportion of cells maintaining membrane integrity and cell surface calreticulin+ cells was analyzed in human PDAC cells by flow cytometry. Virus-treated human PDAC cells showed more than 70% of zombie dye-unlabeled cells maintaining membrane integrity (Figure S3), in which the proportion of cell surface calreticulin+ cells was significantly increased in human PDAC cells, except for Capan-2 cells, after treatment with OBP-702 (Figure S4). In contrast, OBP-301 significantly increased the proportion of cell surface calreticulin+ cells in PK-45H and MIA PaCa-2 cells. These results suggest that p53-armed OBP-702 is superior to OBP-301 in terms of inducing ICD in human PDAC cells with different p53 status.

### OBP-702-induced ICD in human PDAC cells is associated with p53-mediated apoptosis and autophagy

**Figure 4:**
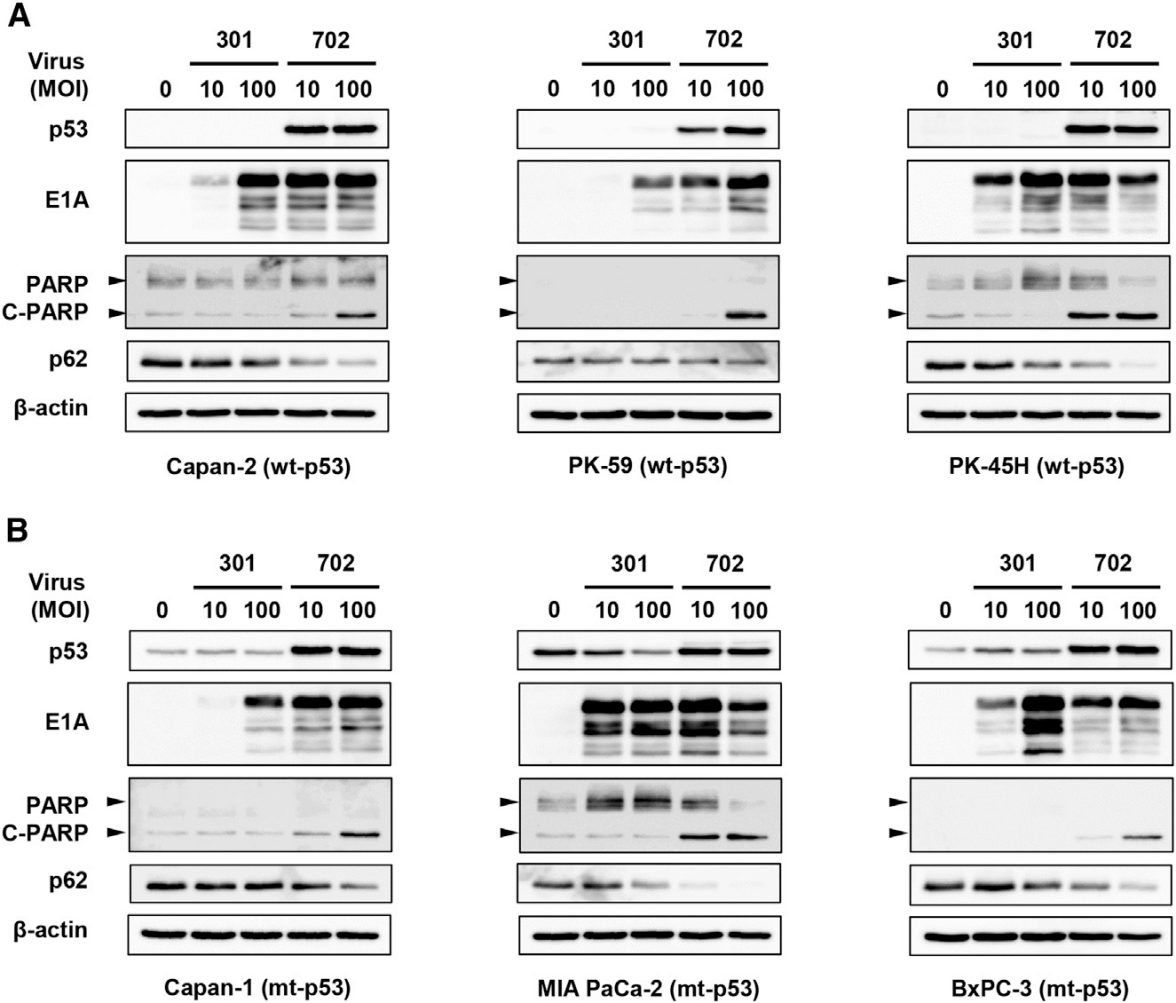
OBP-702 induces p53-mediated apoptosis and autophagy in human PDAC cells (A and B) Human PDAC cells with WT-p53 (A) and mt-p53 (B) were infected with OBP-301 or OBP-702 at the indicated MOI for 48 h. Cell lysates were prepared and subjected to western blot analysis of p53, E1A, PARP, cleaved PARP (C-PARP), and p62 expression. β-Actin was assayed as a loading control. Uninfected (mock-treated) cells were shown as virus-infected cells at an MOI of 0.

To explore the underlying mechanism of the strong OBP-702-induced ICD, we analyzed the expression of p53, adenoviral E1A, cleaved PARP (an apoptosis marker), and p62 (an autophagy marker) proteins in p53-intact and p53-mutant human PDAC cells after viral infection. Western blot analysis demonstrated that OBP-702 increased the expression of p53, E1A, and cleaved PARP in all human PDAC cells, whereas OBP-301 increased the expression of E1A (Figures 4A and 4B). Moreover, OBP-702 decreased the expression of p62 in human PDAC cells to a greater degree than OBP-301 (Figures 4A and 4B). These results suggest that OBP-702-induced ICD in human PDAC cells is associated with p53-mediated induction of apoptosis and autophagy.

### OBP-702 induces more profound ICD than Ad-p53 in human PDAC cells

We previously demonstrated that p53-armed oncolytic adenovirus OBP-702 induces a higher level of p53 expression and apoptosis compared with p53-expressing non-replicative adenovirus Ad-p53 in human cancer cells. ^16^ To investigate the ICD-inducing activity of Ad-p53, human PDAC cells were infected with Ad-p53 for 48 h, and cell viability was assessed using an XTT assay. Among human PDAC cells, BxPC-3 cells were sensitive to and other PDAC cells were resistant to Ad-p53 treatment (Figure S5). The levels of extracellular ATP and HMGB1 were determined using conditioned medium from human PDAC cells after infection with Ad-p53 for 48 h. Ad-p53 significantly increased the release of extracellular ATP and HMGB1 in p53-intact PK-45H cells and p53-mutant MIAPaCa-2 and BxPC-3 cells (Figures S6 and S7). The proportion of zombie dye-unlabeled cells maintaining membrane integrity and cell surface calreticulin+ cells was analyzed in human PDAC cells by flow cytometry. Ad-p53-treated human PDAC cells showed more than 60% of zombie dye-unlabeled cells maintaining membrane integrity, in which the proportion of cell surface calreticulin+ cells was not increased (Figures S8 and S9). Western blot analysis demonstrated that Ad-p53 induced apoptosis only in MIA PaCa-2 cells, although the expression of p53 was increased in all human PDAC cells (Figure S10). These results suggest that p53-armed OBP-702 is superior to Ad-p53 in terms of inducing ICD in human PDAC cells.

### OBP-702 induces ICD in murine PDAC cells more strongly than OBP-301 and Ad-p53

Coxsackie virus and adenovirus receptor (CAR) is a primary receptor for Ad5-based vectors.^19^ CAR-negative cells are also sensitive to Ad5 infection via binding of the virus to the cell surface integrin αvβ5.^20^ As murine PDAC PAN02 cells are frequently used to evaluate the anti-tumor immune response using an immunocompetent mouse model,^21^ we investigated the expression levels of CAR and two integrins (αvβ3 and αvβ5) on the surface of murine PAN02 cells. Flow cytometric analysis demonstrated that PAN02 cells expressed a low level of CAR and high levels of the integrins αvβ3 and αvβ5 (Figure S11). These results suggest that the susceptibility of PAN02 cells to adenovirus infection involves binding of the virus to cell surface integrins rather than CAR.

To evaluate the ability of oncolytic adenoviruses to infect target tumor cells, we previously developed a green fluorescent protein (GFP)-expressing, telomerase-specific, replication-competent OBP-301 variant (OBP-401) (Figure S12A).^22^ To investigate whether OBP-401 induces the expression of GFP in murine PAN02 cells, PAN02 cells were infected with OBP-401 at an MOI of 100 for 24 h. OBP-401 efficiently induced the expression of GFP in PAN02 cells (Figure S12B). These results suggest that PAN02 cells are susceptible to oncolytic adenovirus-mediated *GFP* gene transfer.

**Figure 5:**
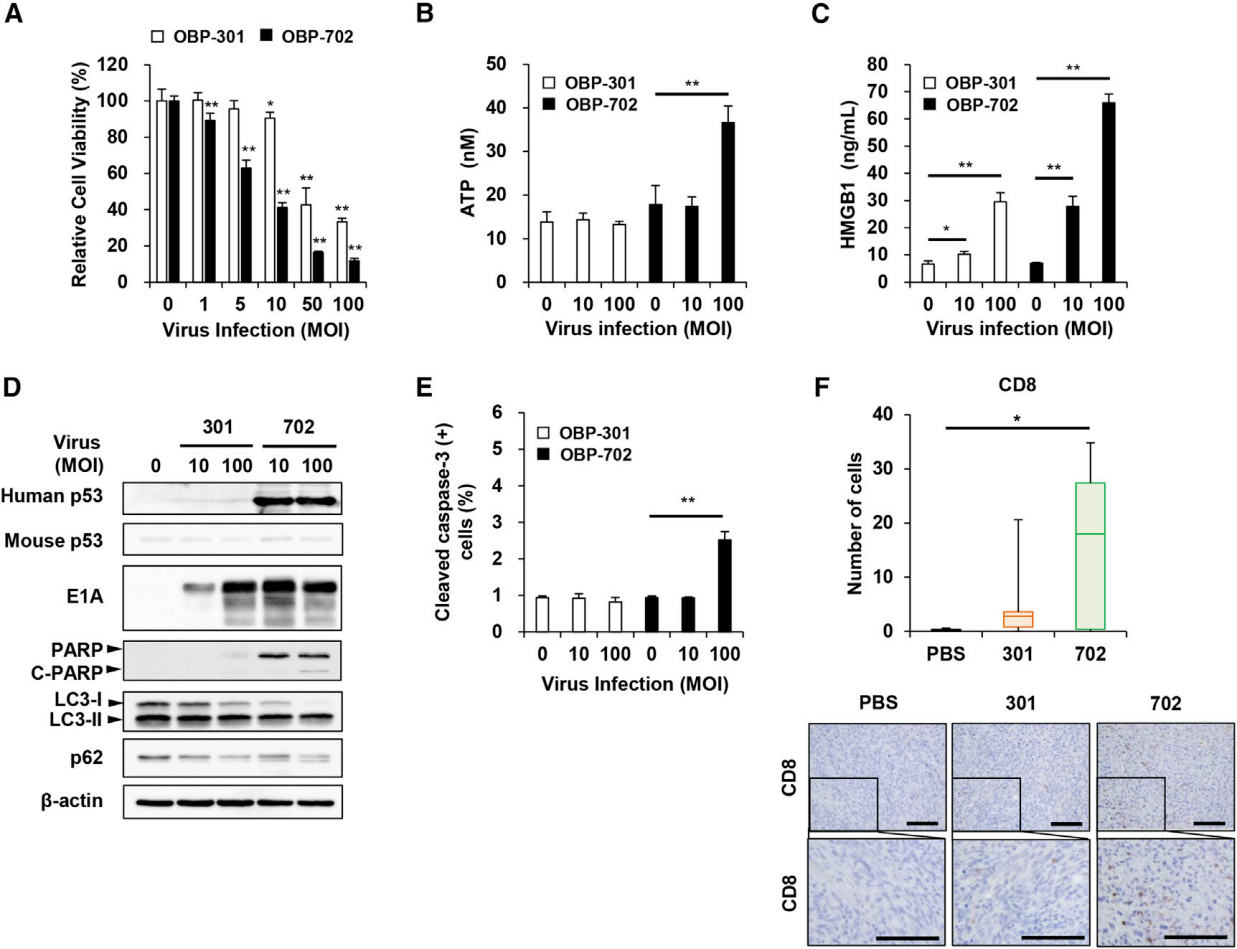
OBP-702 induces immunogenic cell death in murine PDAC cells (A and B) Murine PAN02 cells were treated with OBP-301 or OBP-702 at the indicated MOI for 48 h. Uninfected (mock-treated) cells were shown as virus-infected cells at an MOI of 0. (A) Cell viability was quantified using the XTT assay. Cell viability was calculated relative to that of the mock-infected group, which was set at 100%. Cell viability data are expressed as mean ± SD (n = 5). Student’s t test was used to evaluate the significance of differences. ∗∗p < 0.01 (versus an MOI of 0). (B and C) Supernatants were collected, and the levels of extracellular ATP (B) and HMGB1 (C) were determined using an ENLITEN ATP assay and HMGB1 ELISA kit, respectively. Data are expressed as mean ± SD (n = 3). Student’s t test was used to evaluate the significance of differences. ∗∗p < 0.01 (versus an MOI of 0). (D) Cell lysates were subjected to western blot analysis of human and mouse p53, E1A, PARP, cleaved PARP (C-PARP), LC3, and p62 expression. β-Actin was assayed as a loading control. (E) The percentage of cleaved caspase-3+ cells was quantified by flow cytometry. Data are expressed as mean ± SD (n = 3). Student’s t test was used to evaluate the significance of differences. ∗∗p < 0.01 (versus an MOI of 0). (F) PAN02 cells (2 × 10^6^ cells/site) were inoculated into the flanks of C57BL/6J mice. Mice were then injected intratumorally with 1 × 10^8^ PFUs of OBP-301 or OBP-702 once weekly for three cycles (n = 5). PBS was used as a control. The number of CD8+ T cells was calculated from five different randomly selected fields. Data are expressed as mean ± SD (n = 5). One-way ANOVA followed by the Games-Howell multiple comparison test was used to evaluate the significance of differences. ∗p < 0.05. Representative photographs of immunohistochemical staining for CD8+ T cells in each group. Scale bars, 100 μm.

The observed OBP-401-mediated GFP induction prompted us to investigate the ICD-inducing potential of OBP-301 and OBP-702 in murine PAN02 cells. The viability of PAN02 cells was assessed using the XTT assay after infection with OBP-301 or OBP-702 for 48 h. OBP-702 suppressed the viability of PAN02 cells to a significantly greater degree than OBP-301 (Figure 5A). Levels of extracellular ATP and HMGB1 were then measured using conditioned medium from PAN02 cells infected with OBP-301 or OBP-702 for 48 h. OBP-702 induced significantly greater increases in the levels of extracellular ATP and HMGB1 in PAN02 cells than OBP-301 (Figures 5B and 5C). Virus-treated murine PAN02 cells showed more than 90% of zombie dye-unlabeled cells maintaining membrane integrity, in which the proportion of cell surface calreticulin+ cells was increased after treatment with OBP-702, although there was no significant difference (Figure S13). Western blot analysis demonstrated that OBP-702 increased the expression of human p53, E1A, and cleaved PARP proteins in murine PAN02 cells, whereas the expression of endogenous mouse p53 protein was not increased by OBP-702 (Figure 5D). OBP-301 increased the expression of E1A protein (Figure 5D). Moreover, OBP-702 decreased the expression of p62 protein and increased the conversion of LC3-I to LC3-II in PAN02 cells more strongly than OBP-301 (Figure 5D). Flow cytometric analysis demonstrated that the proportion of cleaved caspase-3+ cells was significantly increased in PAN02 cells after infection with OBP-702 at an MOI of 100 for 48 h (Figure 5E). In contrast, Ad-p53 did not suppress the viability of PAN02 cells (Figure S14A), although the level of extracellular HMGB1 was significantly increased in association with human p53 upregulation (Figures S14B–S14D). These results suggest that OBP-702 induces ICD in murine PDAC cells via the induction of p53-mediated apoptosis and autophagy.

### OBP-702 increases the infiltration of CD8+ T cells in murine PAN02 tumors

The immune-activating effects of OBP-301 and OBP-702 in murine PDAC tumors were analyzed using a syngeneic mouse model of subcutaneous PAN02 tumors. Mice were injected intratumorally with OBP-301, OBP-702, or phosphate-buffered saline (PBS) once weekly for three cycles. Immunohistochemical analysis demonstrated that the number of CD8+ T cells and CD11c+ dendritic cells was significantly increased in tumors treated with OBP-702 but not in tumors treated with OBP-301, compared with control tumors (Figures 5F and S15A). In contrast, there was no significant difference in the number of Foxp3+ T cells (Figure S15A). The Ad5-positive cells were observed in virus-treated PAN02 tumors (Figure S15B). These results suggest that OBP-702 is superior to OBP-301 in terms of inducing the infiltration of cytotoxic T cells in murine PDAC tumors.

### Combined treatment with anti-PD-1 antibody and OBP-702 suppresses the growth of PAN02 tumors via enhancing the infiltration of cytotoxic T cells

**Figure 6:**
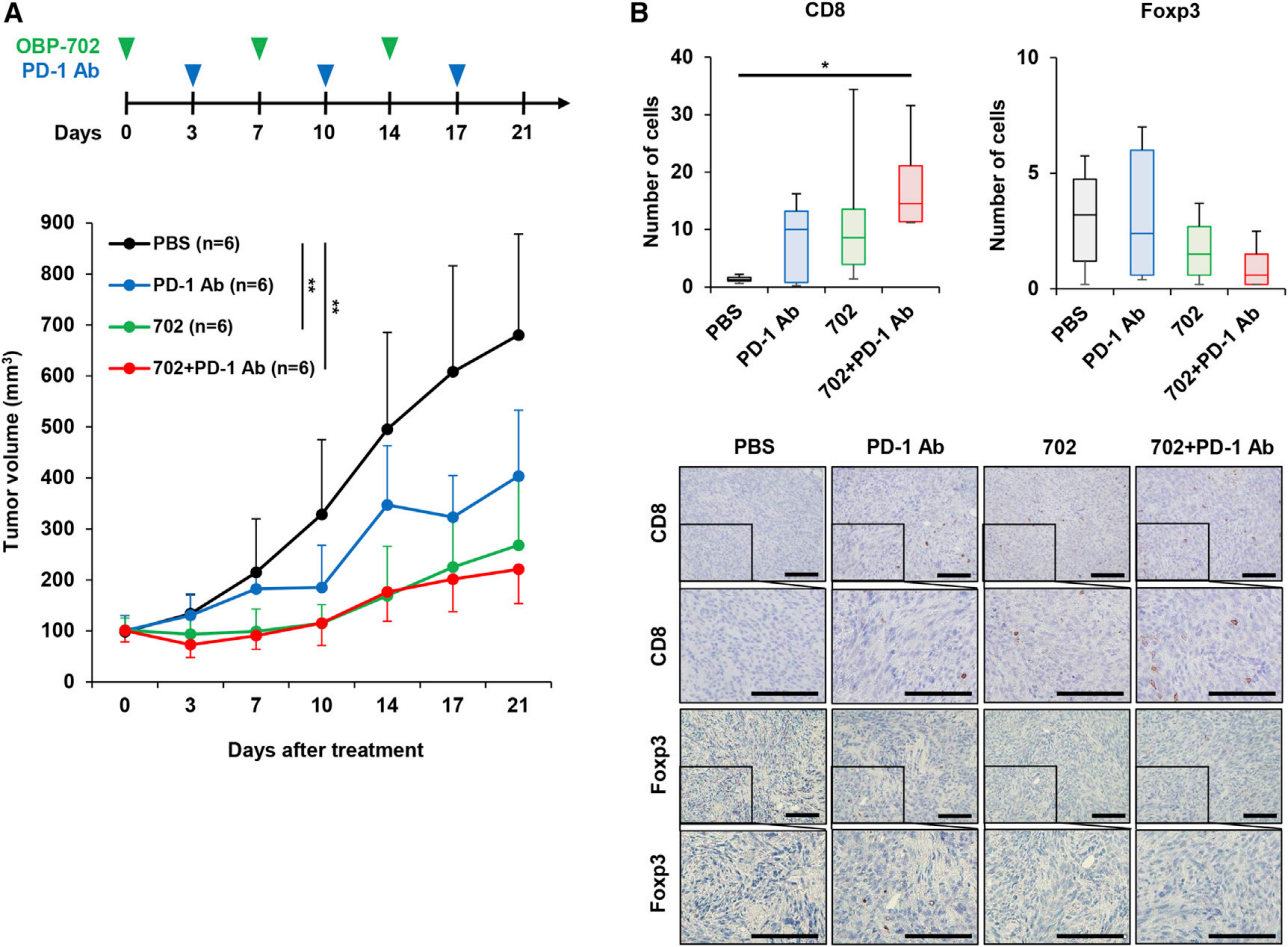
*In vivo* anti-tumor effect of combination therapy with OBP-702 and anti–PD-1 antibody in murine PAN02 tumor model mice (A) PAN02 cells (5 × 10^6^ cells/site) were inoculated into the flanks of C57BL/6J mice. Mice were then injected intratumorally with 1 × 10^8^ PFUs of OBP-702 once weekly for three cycles (green arrowheads). Anti-PD-1 antibody was administered intraperitoneally once weekly for three cycles (blue arrowheads). PBS (black arrowheads) was used as a control. (B) The number of CD8+ and Foxp3+ cells was calculated from five randomly selected fields. Data are expressed as mean ± SD (n = 6). One-way ANOVA followed by the Games-Howell multiple comparison test was used to evaluate the significance of differences. ∗p < 0.05; ∗∗p < 0.01. Representative photographs of immunohistochemical staining for CD8+ and Foxp3+ T cells in each group. Scale bars, 100 μm.

As OBP-702 treatment significantly increased the tumor infiltration of CD8+ T cells (Figure 5F), the anti-tumor effect of combination therapy with PD-1 blockade and OBP-702 was investigated further using the syngeneic mouse model of subcutaneous PAN02 tumors. Mice were injected intratumorally with OBP-702 or PBS once weekly for three cycles. Three days after virus injection, mice were injected intraperitoneally with anti-PD-1 antibody or PBS once weekly for three cycles. Compared with the control group, monotherapy with OBP-702 significantly suppressed the growth of PAN02 tumors, whereas monotherapy with anti-PD-1 antibody did not suppress tumor growth (Figure 6A). Combination therapy with anti-PD-1 antibody and OBP-702 significantly suppressed the growth of PAN02 tumors compared with the control group (Figure 6A). Immunohistochemical analysis demonstrated that the number of CD8+ T cells was significantly increased in tumors treated with combination therapy when compared with the control group (Figure 6B). In contrast, there was no significant difference in the number of Foxp3+ T cells (Figure 6B). These results suggest that OBP-702 has therapeutic potential for enhancing the anti-tumor efficacy of PD-1 blockade in murine PDAC tumors by inducing the infiltration of cytotoxic T cells.

## Discussion

As PDAC tumors are cold and exhibit poor infiltration of cytotoxic T cells, they are typically refractory to immunotherapy with ICIs. Therefore, combination immunogenic therapies that induce ICD are needed to improve the anti-tumor efficacy of ICIs against PDAC tumors. In this study, we demonstrated that a p53-expressing telomerase-specific oncolytic adenovirus, OBP-702, has therapeutic potential for profoundly enhancing ICD in human and murine PDAC cells via the induction of p53-mediated apoptosis and autophagy. Combination therapy with anti-PD-1 antibody and OBP-702 significantly suppressed the growth of PAN02 tumors by enhancing the infiltration of CD8+ T cells. Thus, oncolytic adenovirus-mediated p53 overexpression appears to be a promising anti-tumor strategy for improving the efficacy of ICIs against PDAC tumors by strongly enhancing ICD and the anti-tumor immune response.

Compared with non-armed OBP-301, p53-armed OBP-702 significantly increased the release of extracellular ATP in human PDAC cells (Figure 2). ATP release was significantly greater in p53-intact human PDAC cells than p53-mutant human PDAC cells (Figure 2), suggesting that p53 functions intrinsically to promote the release of ATP by PDAC cells. ATP is primarily generated through several metabolic pathways, including glycolysis and mitochondrial oxidative phosphorylation. Wild-type p53 protein enhances mitochondrial oxidative phosphorylation,^23^ whereas mutant p53 protein suppresses mitochondrial oxidative phosphorylation.^24^ Therefore, p53-mediated activation of mitochondrial oxidative phosphorylation may be involved in the release of ATP from human PDAC cells.

The release of HMGB1 from human PDAC cells was also increased more significantly by p53-armed OBP-702 than non-armed OBP-301 (Figure 3). Of the human PDAC cells with different p53 status examined in this study, p53-mutant BxPC-3 cells released the highest level of HMGB1 after virus infection (Figure 3). HMGB1 is a non-histone protein that predominantly localizes in the cell nucleus. Various cellular stresses, including hypoxia and reactive oxygen species exposure, induce the translocation of HMGB1 from the nucleus to the cytoplasm in damaged cells.^25^ The release of cytoplasmic HMGB1 into the extracellular space is passively induced by damage to the cell membrane.^26^ As p53-mutant BxPC-3 cells were the most sensitive of the PDAC cells to the OBP-702-mediated cytopathic effect (Figure 1), a loss of cellular integrity may be involved in the release of HMGB1 from OBP-702-treated PDAC cells.

The p53-mediated induction of apoptosis and autophagy in human and murine PDAC cells observed in the present study was associated with OBP-702-induced release of ATP and HMGB1 (Figures 4 and 5). Both apoptosis and autophagy are thought to regulate the cellular release of ATP and HMGB1. For example, caspase-dependent activation of the pannexin-1 channel facilitates the release of ATP from apoptotic cells.^27^^,^^28^ Release of ATP is suppressed in autophagy-deficient cancer cells treated with chemotherapy.^29^ The release of HMGB1 is also associated with autophagy,^30^ apoptosis,^31^ and necrosis.^32^ In contrast, activation of p53 expression induces the release of HMGB1 from normal cells.^33^^,^^34^ p53 protein interacts with HMGB1 to modulate apoptosis and autophagy.^35^ Thus, p53-mediated induction of apoptosis and autophagy may cooperatively promote the release of extracellular ATP and HMGB1 from PDAC cells.

It is worth noting that OBP-702 efficiently induced ICD in murine PAN02 cells in the present study, despite low CAR expression (Figures 5 and S11). We recently demonstrated that the therapeutic potential of an RGD-fiber-modified OBP-301 variant against murine PAN02 cells was enhanced by increasing the binding affinity for integrins.^11^ However, Lyle et al. suggested that the sensitivity of CAR-negative cells to adenovirus infection involves binding of the virus to integrin αvβ5.^20^ As CAR-negative murine PAN02 cells exhibited high integrin αvβ5 expression and high sensitivity to infection with OBP-301 and OBP-702, murine PAN02 cells would be a useful model for evaluating the anti-tumor effect and immune-activating ability of oncolytic adenoviruses in immunocompetent mice.

OBP-702 was generated by inserting the human p53 expression cassette into the E3 region of OBP-301 (Figure S1). Several reports have shown that adenovirus E3 protein modulates the efficacy of oncolytic adenovirus. Suzuki et al. demonstrated that adenovirus E3 promotes the replication of oncolytic adenovirus, leading to the enhancement of cytopathic effect.^36^ Wang et al. showed that E3-intact adenovirus induces more profound anti-tumor immunity with tumor infiltration of CD8+ T cells than E3-deleted adenovirus.^37^ In contrast, Hibma et al. demonstrated that partially E3-deleted adenovirus dl309 induces more profound cytopathic effect than Ad5 in CAR-negative cancer cells by increasing apoptosis.^38^ Moreover, Hastie et al. showed that p53 transgene expression inhibits the antiviral signaling in human PDAC cells,^39^ suggesting the p53-mediated enhancement of viral replication. Thus, the crosstalk between modification of adenovirus E3 region and p53 upregulation may play a crucial role in the underlying mechanism of OBP-702-mediated anti-tumor effect.

Combination treatment with OBP-702 significantly increased the anti-tumor effect of PD-1 blockade in murine PAN02 tumors (Figure 6). The number of tumor-infiltrating CD8+ T cells was significantly increased in combination therapy (Figure 6), suggesting that OBP-702 has therapeutic potential to promote the anti-tumor immune response against PDAC tumors. However, there are some limitations to the use of murine PAN02 cells for evaluating the anti-tumor effect of combination therapies against PDAC tumors. PAN02 cells reportedly lack mutant *KRAS* gene and a stromal microenvironment.^21^ Oncogenic KRAS activation and cancer-associated fibroblasts have been shown to play an important role in generating an immunosuppressive microenvironment.^40^^,^^41^ Therefore, further studies to evaluate the anti-tumor effect of combination therapy with OBP-702 and PD-1 blockade using KRAS-mutant murine PDAC tumors with massive stroma are warranted. Moreover, we used a subcutaneous PAN02 tumor model in this study. An orthotopic PAN02 tumor model would be warranted to evaluate the anti-tumor effect and survival benefit in tumor-bearing mice after treatment with combination therapy of OBP-702 and PD-1 blockade.

Recent accumulating evidences have suggested the utility of Syrian hamster as immunocompetent and replication-permissive animal model for evaluating the anti-tumor effect of oncolytic adenovirus.^42^ Spencer et al. established Syrian hamster PDAC cell lines SHPC6 that are transplantable to develop subcutaneous tumors, lung metastasis, and intraperitoneal metastasis.^43^ SHPC6 tumor models are useful tools to evaluate the anti-tumor effect of oncolytic adenovirus INGN 007.^43^ Although the cytopathic activity of telomerase-specific replication-competent OBP-702 against Syrian hamster PDAC cells remains unclear, PDAC tumor models using Syrian hamster would be a more suitable model to evaluate the anti-tumor effect and safety of combination therapy with OBP-702 and PD-1 blockade.

In conclusion, we demonstrated that p53-armed telomerase-specific oncolytic adenovirus OBP-702 enhances the anti-tumor efficacy of PD-1 blockade in PDAC tumors via p53-mediated ICD induction and promotion of cytotoxic T cell infiltration. Combination therapy with PD-1 blockade and OBP-702 is a promising anti-tumor strategy for converting cold PDAC tumors to hot PDAC tumors. Thus, oncolytic adenovirus-mediated p53 overexpression represents a novel therapeutic option for the treatment of immunotherapy-refractory PDAC tumors.

## Materials and methods

### Cell lines

Four human PDAC cell lines (Capan-1, Capan-2, MIA PaCa-2, BxPC-3) were obtained from the American Type Culture Collection (Manassas, VA, USA). Two other human PDAC cell lines (PK-45H, PK-59) were obtained from the Cell Resource Center for Biomedical Research, Institute of Development, Aging and Cancer, Tohoku University (Sendai, Japan). Capan-2, PK-59, and PK-45H cells express wild-type p53 protein and Capan-1 (159 Val), MIA PaCa-2 (248 Trp), and BxPC-3 (220 Cys) cells express mutant p53 protein, as shown by previous report.^44^ The murine PDAC cell line PAN02 was obtained from the US National Cancer Institute (Frederick, MD, USA). PAN02 cells express wild-type p53 protein, as shown by previous report.^45^ Cells were cultured for no longer than 5 months following resuscitation. The authors did not authenticate the cell lines. Capan-1 cells were maintained in Iscove’s modified Dulbecco’s medium supplemented with 20% fetal bovine serum (FBS). Capan-2 cells were maintained in McCoy’s 5A medium supplemented with 10% FBS. MIA PaCa-2 cells were maintained in Dulbecco’s Modified Eagle’s Medium supplemented with 10% FBS. BxPC-3, PK-45H, PK-59, and PAN02 cells were maintained in RPMI1640 medium supplemented with 10% FBS. All media were supplemented with 100 U/mL penicillin and 100 μg/mL streptomycin. All cells were routinely maintained at 37°C in a humidified atmosphere with 5% CO_2_.

### Recombinant adenoviruses

The recombinant telomerase-specific replication-competent adenovirus OBP-301 (Suratadenoturev), in which the promoter element of the *hTERT* gene drives expression of the *E1A* and *E1B* genes, was previously constructed using the adenovirus serotype 5 (Ad5) genome (Figure S1).^7^^,^^8^ For OBP-301-mediated tumor-specific induction of exogenous p53 expression, we further generated OBP-702 by inserting a human wild-type *p53* gene expression cassette derived from the Egr-1 promoter into the E3 region of the OBP-301 genome (Figure S1).^16^^,^^17^ Ad-p53 was used as a p53-expressing non-replicative adenovirus (Figure S1). Recombinant viruses were purified by ultracentrifugation using cesium chloride step gradients, and viral titer was determined by a plaque-forming assay using 293 cells. All viruses were stored at −80°C.

### ICI

Anti-mouse PD-1 antibody (clone 4H2) was obtained from Ono Pharmaceutical (Osaka, Japan).

### Cell viability assay

PK-45H, Capan-1, MIA PaCa-2, BxPC-3, and PAN02 cells were seeded in 96-well plates at a density of 1 × 10^3^ cells/well, whereas Capan-2 and PK-59 cells were seeded at a density of 5 × 10^3^ cells/well in 96-well plates, 24 h before viral infection. Murine and human PDAC cells were infected with OBP-301, Ad-p53, or OBP-702 at an MOI of 0, 1, 5, 10, 50, or 100 PFU/cell for 48 and 120 h (n = 5). Cell viability was determined using a Cell Proliferation kit II (Roche Molecular Biochemicals, Indianapolis, IN, USA) according to the manufacturer’s protocol.

### DAMP analysis

Cells were seeded in 6-well plates at a density of 2 × 10^5^ cells/well 24 h before viral infection. Murine and human PDAC cells were infected with OBP-301, Ad-p53, or OBP-702 at an MOI of 0, 10, and 100 plaque-forming units (PFU)/cell for 48 h (n = 3). Supernatant was collected and analyzed using an ENLITEN ATP assay (Promega, Madison, WI, USA) and HMGB1 ELISA kit II (Shino-Test, Kanagawa, Japan).

### Western blotting

Cells were seeded in a 100-mm dish at a density of 1 × 10^6^ cells/dish 24 h before viral infection. Murine and human PDAC cells were infected with OBP-301, Ad-p53, or OBP-702 at an MOI of 0, 10, and 100 PFU/cell for 48 h. Whole-cell lysates were prepared in lysis buffer (50 mM Tris-HCl [pH 7.4], 150 mM NaCl, 1% Triton X-100) containing a protease inhibitor cocktail (Complete Mini; Roche, Indianapolis, IN, USA). Proteins were electrophoresed on 8%–10% sodium dodecyl sulfate-polyacrylamide gels and then transferred onto polyvinylidene difluoride membranes (Hybond-P; GE Health Care, Buckinghamshire, UK). The membranes were blocked with Blocking-One (Nacalai Tesque, Kyoto, Japan) at room temperature for 30 min. The membranes were probed with the following primary antibodies: mouse anti-human p53 monoclonal antibody (mAb) (18032; Cell Signaling Technology, Beverly, MA, USA); rabbit anti-mouse p53 mAb (32532; Cell Signaling Technology); mouse anti-Ad5 E1A mAb (554155; BD Bioscience, Franklin Lakes, NJ, USA); rabbit anti-poly (ADP-ribose) polymerase (PARP) polyclonal antibody (pAb) (9542; Cell Signaling Technology); rabbit anti-LC3 mAb (12741; Cell Signaling Technology); rabbit anti-p62 mAb (5114; Cell Signaling Technology); and mouse anti-β-actin mAb (A5441; Sigma-Aldrich, St. Louis, MO, USA). The following secondary antibodies were used: horseradish peroxidase-conjugated antibodies against mouse IgG (NA931; GE Healthcare) or rabbit IgG (NA934; GE Healthcare). Immunoreactive bands on the blots were visualized using enhanced chemiluminescence substrate (ECL Prime; GE Healthcare) (Figures S16–S21).

### Flow cytometric analysis

Cells were seeded in 6-well plates at a density of 2 × 10^5^ cells/well 24 h before viral infection. Murine PAN02 cells were infected with OBP-301 or OBP-702 at an MOI of 0, 10, and 100 PFU/cell for 48 h (n = 3). To analyze the proportion of cleaved caspase-3+ cells, cells were stained using PE active caspase-3 apoptosis kit (550914; BD Biosciences, San Jose, CA, USA) according to the manufacturer’s protocol. The percentage of cleaved caspase-3+ cells was analyzed using a FACSLyric system (BD Biosciences).

### *In vivo* subcutaneous PAN02 tumor model

Animal experimental protocols were approved by the Ethics Review Committee for Animal Experimentation of Okayama University School of Medicine. To compare the immune-stimulating effect of OBP-301 and OBP-702 in PAN02 tumors, PAN02 cells (10^6^ cells per site) were subcutaneously inoculated into the flank of 6-week-old female C57BL/6J mice (CLEA Japan, Tokyo, Japan). Palpable tumors developed within 14 days and were permitted to grow to approximately 5–6 mm in diameter. A 50-μL volume of solution containing OBP-301 or OBP-702 at a dose of 1 × 10^8^ PFU or PBS was injected intratumorally once weekly for three cycles. In contrast, to evaluate the anti-tumor effect of combination therapy with OBP-702 and PD-1 blockade, a 100-μL volume of solution containing anti-PD-1 antibody (first: 20 mg/kg; second/third: 10 mg/kg) or PBS was administered intraperitoneally once weekly for three cycles. Tumor volume was monitored twice weekly after virus infection. Tumor volume was estimated using the following formula: tumor volume (mm^3^) = *a* × *b*^2^ × 0.5, where *a* represents the longest diameter and *b* represents the shortest diameter.

### Immunohistochemistry

Subcutaneous tumors were fixed in 10% neutralized formalin and embedded in paraffin blocks. Tissue sections (4 μm) were deparaffinized in xylene and rehydrated in a graded ethanol series. After blocking endogenous peroxidases by incubation with 3% H_2_O_2_ for 10 min, the samples were boiled in citrate buffer or EDTA buffer for 14 min in a microwave oven for antigen retrieval. Samples were incubated with primary antibodies for 1 h at room temperature or overnight at 4°C and then with peroxidase-conjugated secondary antibody for 30 min at room temperature. Rat mAbs against CD8 (14-0808-82; eBioscience, San Diego, CA, USA) and Foxp3 (14-5773; eBioscience) and rabbit mAbs against CD11c (97585; Cell Signaling Technology) and Ad5 (ab6982; Abcam, Cambridge, UK) were used as primary antibodies. After staining with 3,3-diaminobenzidine for signal generation and counterstaining with Mayer’s hematoxylin, samples were dehydrated and mounted onto coverslips. The number of cells expressing CD8, Foxp3, and CD11c, which is indicative of cytotoxic T lymphocytes, regulatory T lymphocytes, and dendritic cells, respectively, was determined by counting positive cells in five different randomly selected fields. All sections were analyzed under a light microscope.

### Statistical analysis

Data are expressed as the mean ± SD. The Student’s t test was used to evaluate the significance of differences between two groups. One-way ANOVA followed by the Games-Howell multiple comparison procedure was used to evaluate the significance of differences between more than two groups. Data were analyzed using SPSS Statistics v.26 (SPSS, Chicago, IL, USA). Statistical significance was defined as a p value of less than 0.05.

### Data availability statement

All data are available in the main text or supplemental information.

## Supplemental information

Document S1. Supplemental materials and methods and Figures S1–S21

Document S2. Article plus supplemental information
