# Supplementary material for: Oncolytic virus-mediated p53 overexpression promotes immunogenic cell death and efficacy of PD-1 blockade in pancreatic cancer
Source: Mol Ther Oncolytics. 2022 Sep 13;27:3–13. doi: 10.1016/j.omto.2022.09.003 (PMC9513735; doi:10.1016/j.omto.2022.09.003)
Supplement: Document S1. Supplemental materials and methods and Figures S1–S21 [file mmc1.pdf]

OMTO, Volume *27*

Supplemental information

Oncolytic virus-mediated p53 overexpression promotes immunogenic cell death and efficacy of PD-1 blockade in pancreatic cancer

Hiroyuki Araki, Hiroshi Tazawa, Nobuhiko Kanaya, Yoshinori Kajiwara, Motohiko Yamada, Masashi Hashimoto, Satoru Kikuchi, Shinji Kuroda, Ryuichi Yoshida, Yuzo Umeda, Yasuo Urata, Shunsuke Kagawa, and Toshiyoshi Fujiwara

Supplementary Materials and Methods

Flow cytometric analysis

Cells were seeded in 6-well plates at a density of 2×10^5 cells/well 24 h before viral infection. Murine and human PDAC cells were infected with OBP-301, Ad-p53 or OBP-702 at a MOI of 0, 10, and 100 plaque-forming units (PFU)/cell for 48 h (n = 3). The proportion of cells maintaining membrane integrity was analyzed using Zombie NIR Fixable Viability Kit (423105; BioLegend, San Diego, CA, USA) according to the manufacturer's protocol. To analyze the proportion of cell surface calreticulin+ cells, cells were incubated with mouse anti-calreticulin mAb (ab22683; Abcam, Cambridge, UK) for 60 min on ice. Isotype IgG was used as control IgG. In contrast, to analyze the expression of CAR and integrins $\alpha v\beta 3$ and $\alpha v\beta 5$ in PAN02 cells, uninfected cells were incubated with mouse monoclonal anti-CAR antibody (RmcB; Upstate, Lake Placid, NY, USA), rabbit polyclonal anti-integrin $\alpha v\beta 3$ antibody (bs-1310R; Bioss Antibodies, Woburn, MA, USA), or rabbit anti-polyclonal anti-integrin $\alpha v\beta 5$ antibody (bs-1356R; Bioss Antibodies) for 60 min on ice. The cells were then labeled with fluorescent isothiocyanate-conjugated rabbit anti-mouse IgG as secondary antibody (A16161; Invitrogen, Carlsbad, CA, USA) or Alexa Fluor 647–conjugated goat anti-rabbit IgG as secondary antibody (A-21245; Invitrogen) for 30 min and analyzed using a FACSLyric system (BD Biosciences). The mean fluorescence intensity (MFI) was determined by calculating the difference between the MFI of antibody-treated and isotype control IgG–treated cells.

Recombinant adenoviruses

OBP-401 is a telomerase-specific replication-competent adenovirus, in which the *hTERT* gene promoter drives expression of the *E1A* and *E1B* genes that are linked to an internal ribosome entry site (IRES) and in which the green fluorescent protein (*GFP)* gene is inserted into the E3 region of the genome under a cytomegalovirus (CMV) promoter.

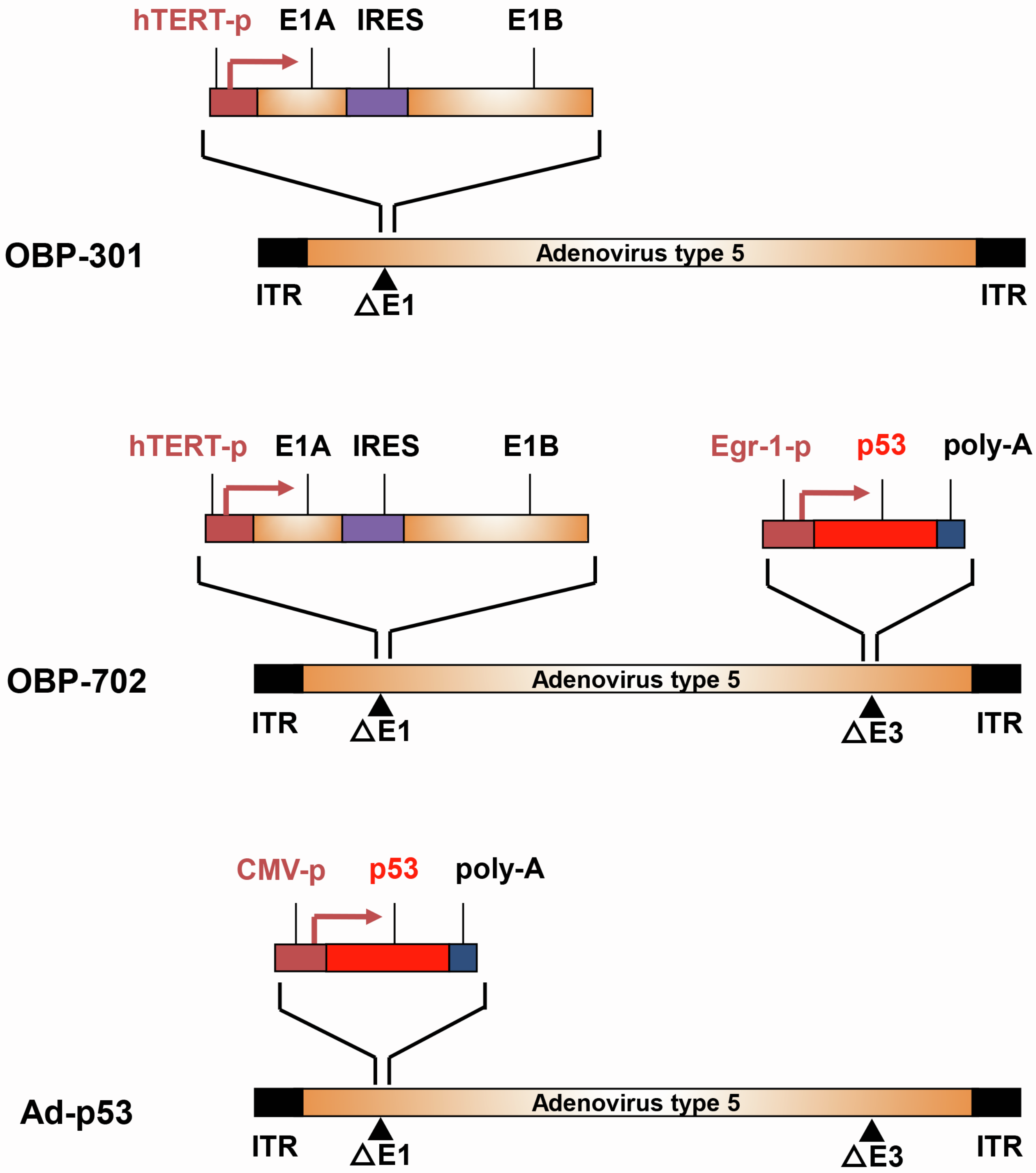

Figure S1. Schematic diagrams of the structures of OBP-301, OBP-702, and Ad-p53.

OBP-301 (suratadenoturev) is a telomerase-specific replication-competent oncolytic adenovirus, in which the hTERT promoter drives expression of the *E1A* and *E1B* genes that are linked with an IRES. In contrast, OBP-702 is a p53-expressing OBP-301 variant, in which a human wild-type *p53* gene expression cassette driven by the Egr-1 promoter is inserted into the E3 region of OBP-301. Ad-p53 is a p53-expressing non-replicative adenovirus, in which a human wild-type *p53* gene expression cassette driven by the CMV promoter is inserted into the E1 region of the Ad5 genome and E3 region is deleted.

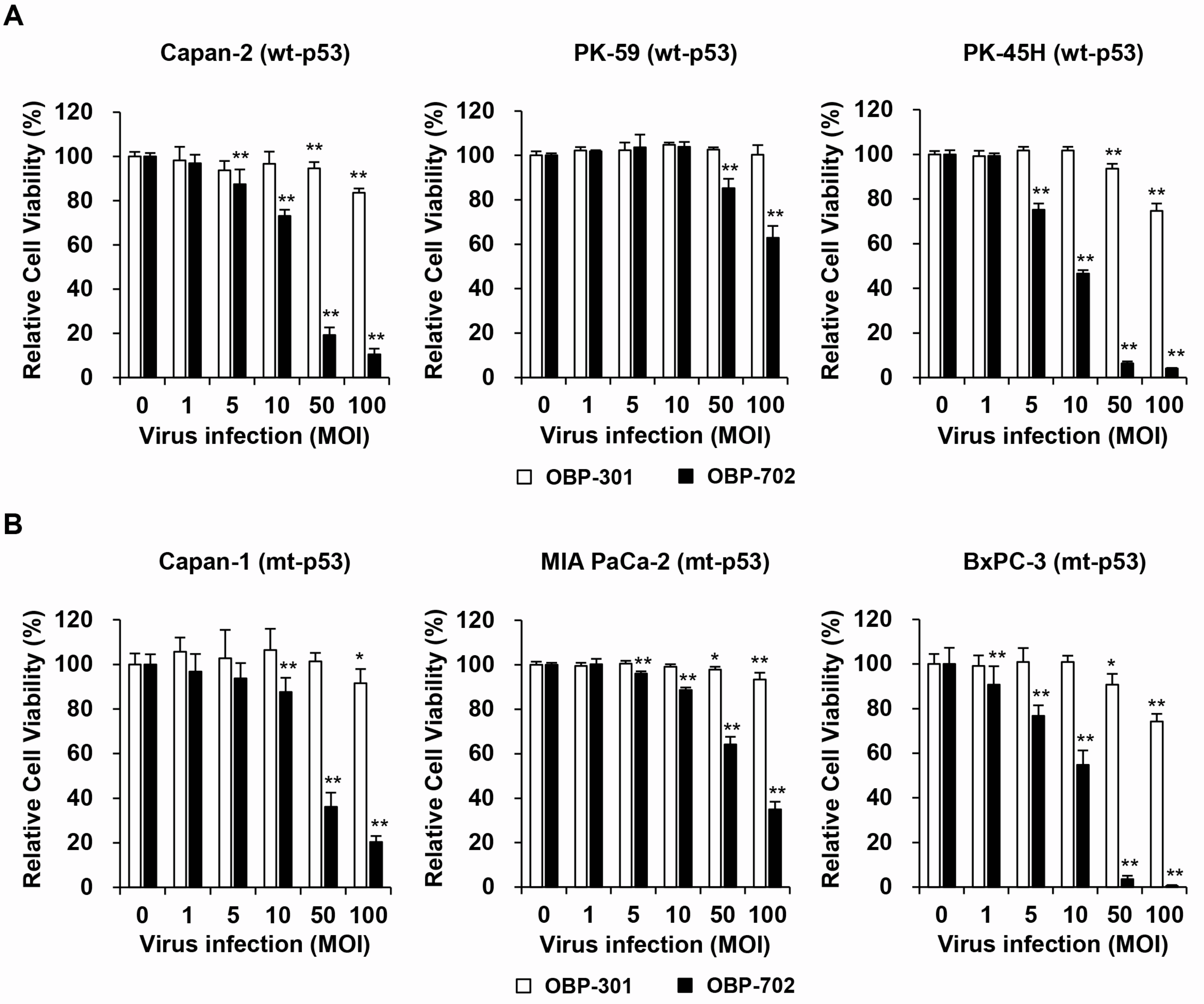

Figure S2. *In vitro* cytopathic effect of OBP-301 and OBP-702 against human PDAC cells.

A, **B**, Human PDAC cells with wild-type p53 (wt-p53) (**A**) and mutant p53 (mt-p53) (**B**) were treated with OBP-301, or OBP-702 at the indicated MOI for 120 h. Cell viability was quantified using the XTT assay. Uninfected (mock-infected) cells were shown as virus-infected cells at an MOI of 0. Cell viability was calculated relative to that of the mock-infected group, which was set at 100%. Cell viability data are expressed as mean ± SD (n = 5). Student's *t* test was used to evaluate the significance of differences. *, $P < 0.05$; **, $P < 0.01$ (versus an MOI of 0).

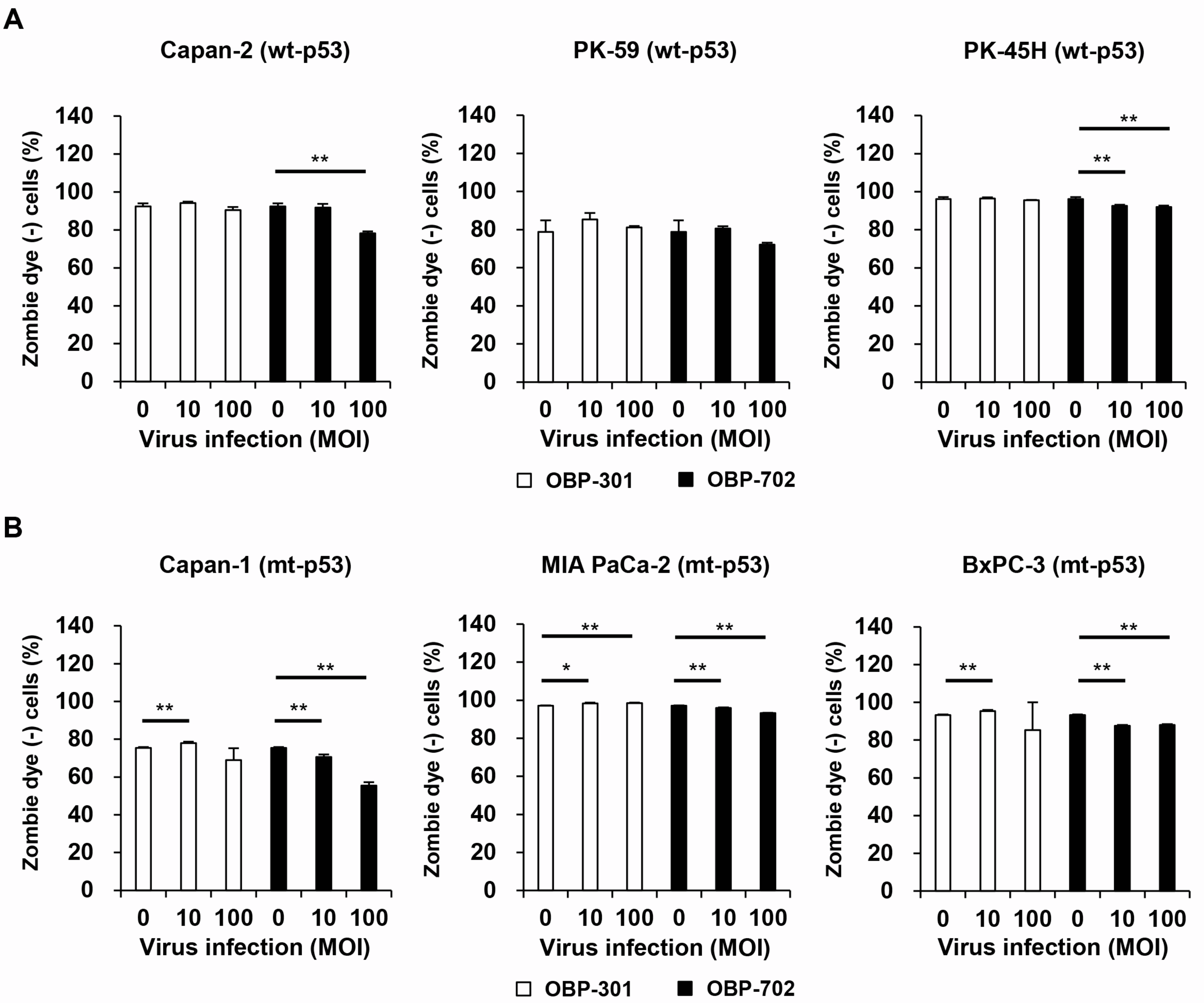

Figure S3. Proportion of zombie dye-unlabeled cells maintaining membrane integrity in human PDAC cells infected with OBP-301 and OBP-702 for 48 h.

A, **B**, Human PDAC cells with wild-type p53 (wt-p53) (**A**) and mutant p53 (mt-p53) (**B**) were treated with OBP-301, or OBP-702 at the indicated MOI for 48 h. The percentage of zombie dye-unlabeled cells maintaining membrane integrity was quantified by flow cytometry. Uninfected (mock-infected) cells were shown as virus-infected cells at an MOI of 0. Data are expressed as mean ± SD (n = 3). Student's *t* test was used to evaluate the significance of differences. *, $P < 0.05$; **, $P < 0.01$ (versus an MOI of 0).

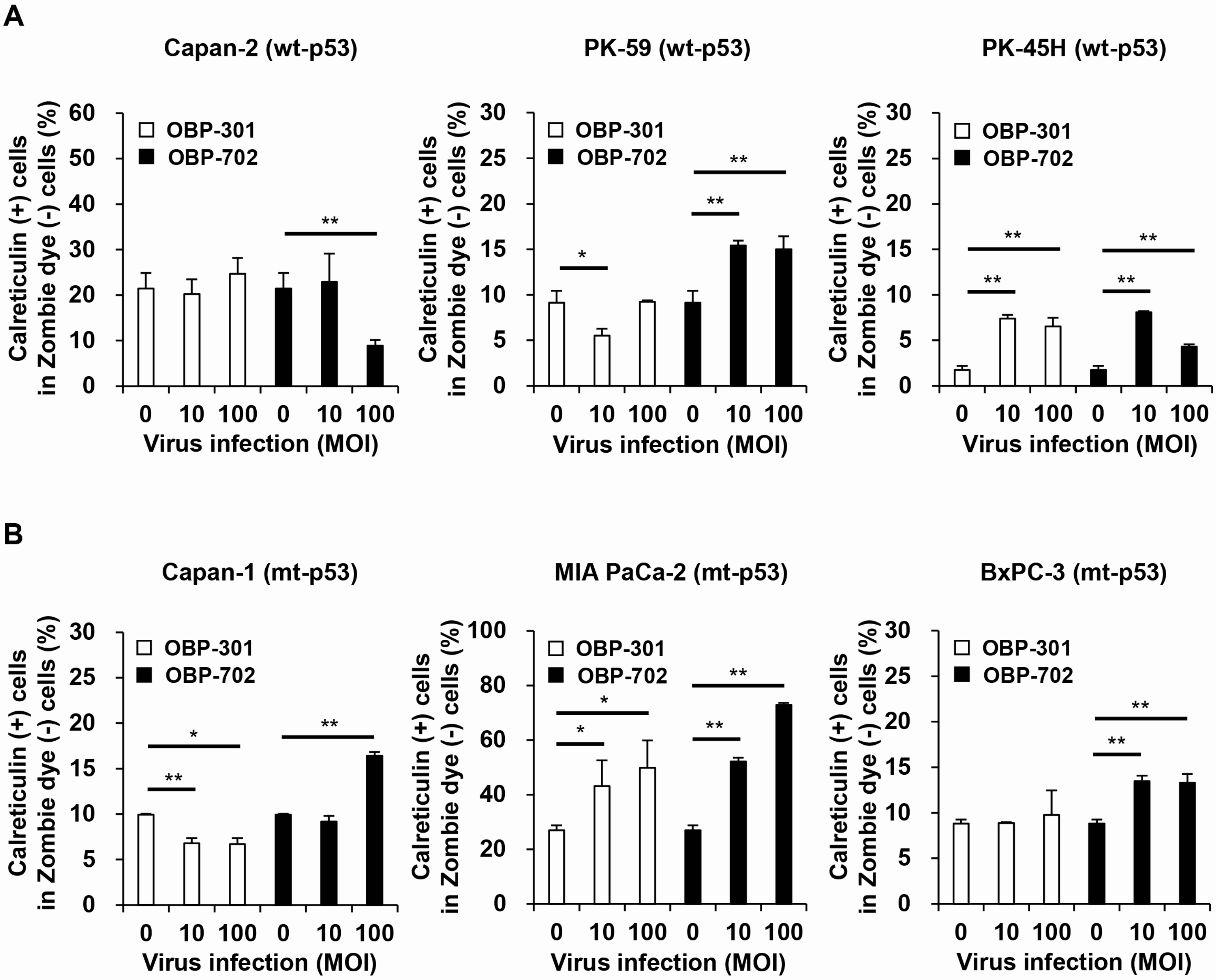

Figure S4. Proportion of calreticulin+ cells in human PDAC cells infected with OBP-301 and OBP-702 for 48 h.

A, **B**, Human PDAC cells with wild-type p53 (wt-p53) (**A**) and mutant p53 (mt-p53) (**B**) were treated with OBP-301, or OBP-702 at the indicated MOI for 48 h. The percentage of calreticulin+ cells among zombie dye-unlabeled cells maintaining membrane integrity was quantified by flow cytometry. Uninfected (mock-infected) cells were shown as virus-infected cells at an MOI of 0. Data are expressed as mean ± SD (n = 3). Student's *t* test was used to evaluate the significance of differences. *, $P < 0.05$; **, $P < 0.01$ (versus an MOI of 0).

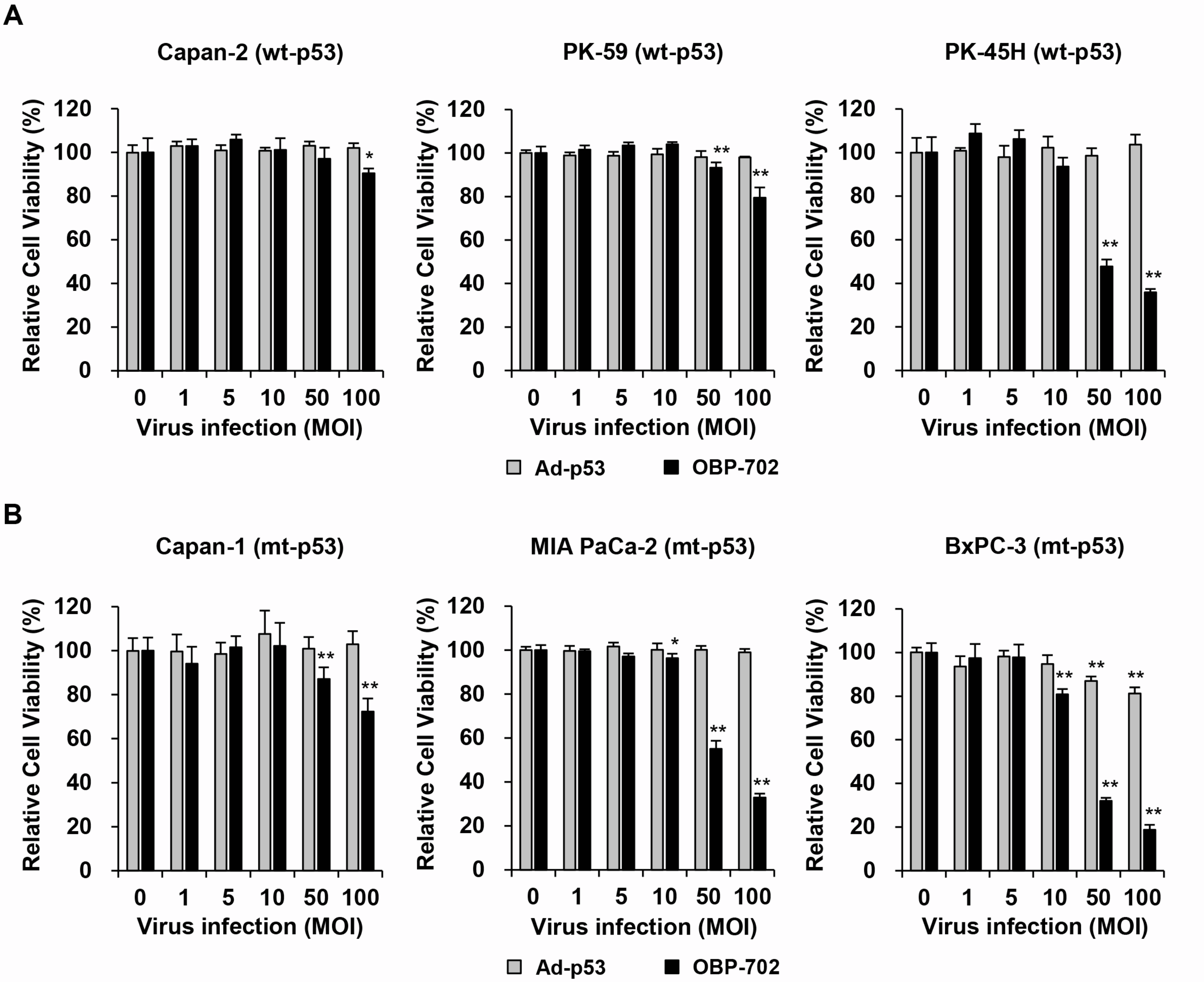

Figure S5. ***In vitro*** **cytopathic effect of Ad-p53 and OBP-702 against human PDAC cells.**

A, **B**, Human PDAC cells with wild-type p53 (wt-p53) (**A**) and mutant p53 (mt-p53) (**B**) were treated with Ad-p53 or OBP-702 at the indicated MOI for 48 h. Cell viability was quantified using the XTT assay. Uninfected (mock-infected) cells were shown as virus-infected cells at an MOI of 0. Cell viability was calculated relative to that of the mock-infected group, which was set at 100%. Cell viability data are expressed as mean ± SD (n = 5). Student's *t* test was used to evaluate the significance of differences. *, $P < 0.05$; **, $P < 0.01$ (versus an MOI of 0).

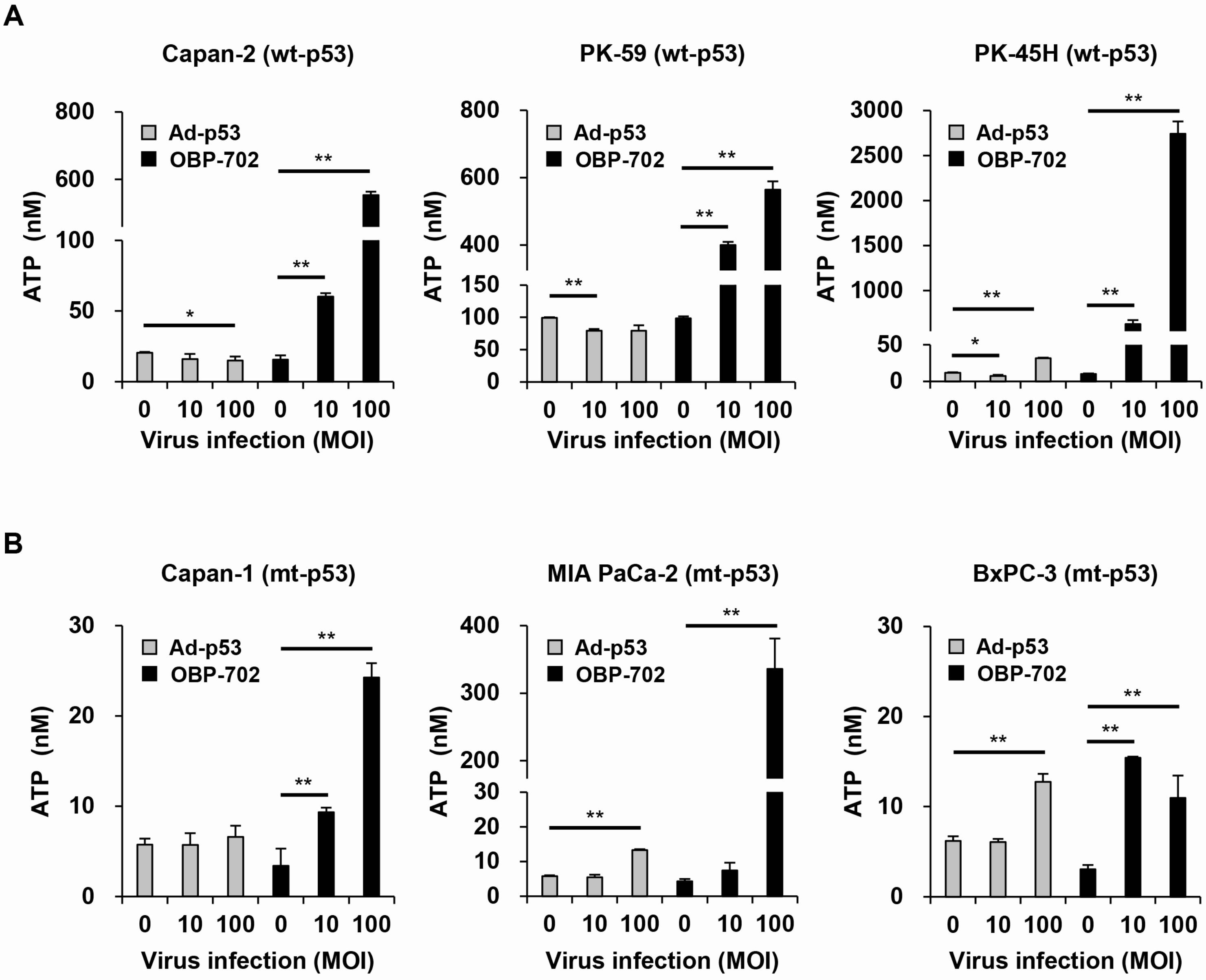

Figure S6. Effect of Ad-p53 and OBP-702 in the release of extracellular ATP in human PDAC cells.

A, **B**, Human PDAC cells with wild-type p53 (wt-p53) (**A**) and mutant p53 (mt-p53) (**B**) were treated with Ad-p53 or OBP-702 at the indicated MOI for 48 h. Supernatants were collected, and the level of extracellular ATP was determined using an ENLITEN ATP assay. Uninfected (mock-infected) cells were shown as virus-infected cells at an MOI of 0. Data are expressed as mean ± SD (n = 3). Student's *t* test was used to evaluate the significance of differences. *, $P < 0.05$; **, $P < 0.01$ (versus an MOI of 0).

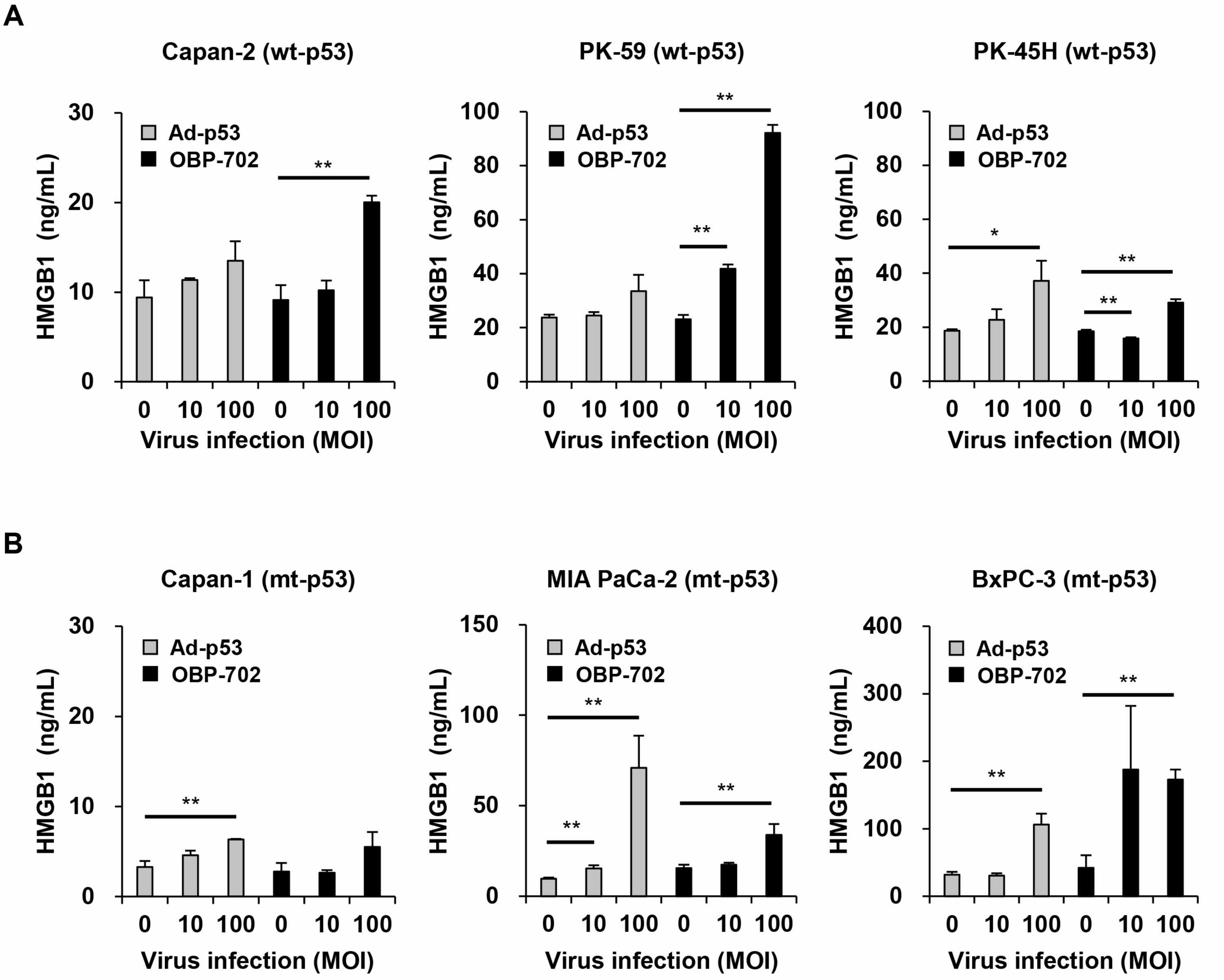

Figure S7. Effect of Ad-p53 and OBP-702 in the release of extracellular HMGB1 in human PDAC cells.

A, **B**, Human PDAC cells with wild-type p53 (wt-p53) (**A**) and mutant p53 (mt-p53) (**B**) were treated with Ad-p53 or OBP-702 at the indicated MOI for 48 h. Supernatants were collected, and the level of extracellular HMGB1 was determined using an HMGB1 ELISA. Uninfected (mock-infected) cells were shown as virus-infected cells at an MOI of 0. Data are expressed as mean ± SD (n = 3). Student's *t* test was used to evaluate the significance of differences. *, $P < 0.05$; **, $P < 0.01$ (versus an MOI of 0).

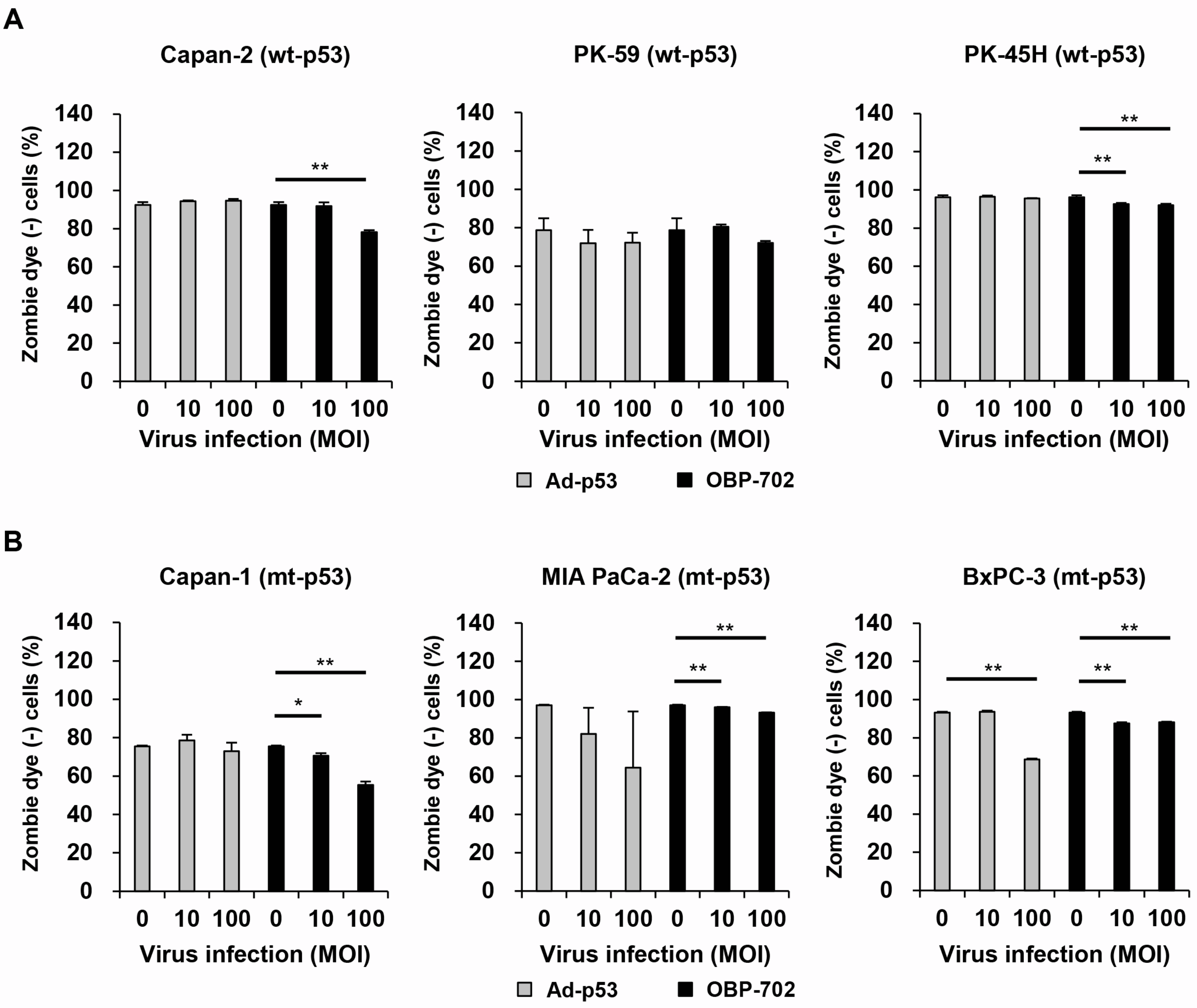

Figure S8. Proportion of zombie dye-unlabeled cells maintaining membrane integrity in human PDAC cells infected with Ad-p53 and OBP-702 for 48 h.

A, **B**, Human PDAC cells with wild-type p53 (wt-p53) (**A**) and mutant p53 (mt-p53) (**B**) were treated with Ad-p53 or OBP-702 at the indicated MOI for 48 h. The percentage of zombie dye-unlabeled cells maintaining membrane integrity was quantified by flow cytometry. Uninfected (mock-infected) cells were shown as virus-infected cells at an MOI of 0. Data are expressed as mean ± SD (n = 3). Student's *t* test was used to evaluate the significance of differences. *, $P < 0.05$; **, $P < 0.01$ (versus an MOI of 0).

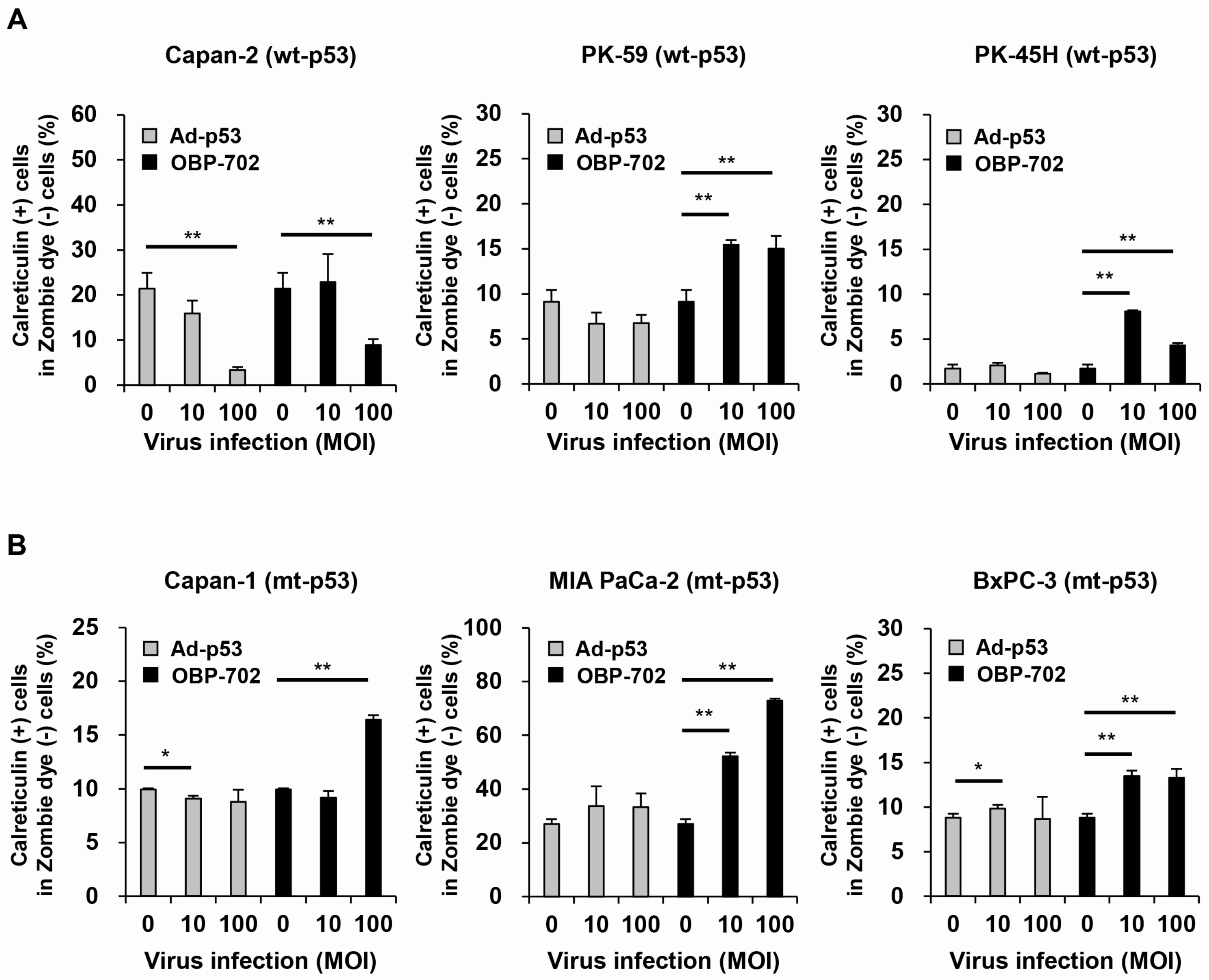

Figure S9. Proportion of calreticulin+ cells in human PDAC cells infected with Ad-p53 and OBP-702 for 48 h.

A, **B**, Human PDAC cells with wild-type p53 (wt-p53) (**A**) and mutant p53 (mt-p53) (**B**) were treated with Ad-p53 or OBP-702 at the indicated MOI for 48 h. The percentage of calreticulin+ cells among zombie dye-unlabeled cells maintaining membrane integrity was quantified by flow cytometry. Uninfected (mock-infected) cells were shown as virus-infected cells at an MOI of 0. Data are expressed as mean ± SD (n = 3). Student's *t* test was used to evaluate the significance of differences. *, *P* < 0.05; **, *P* < 0.01 (versus an MOI of 0).

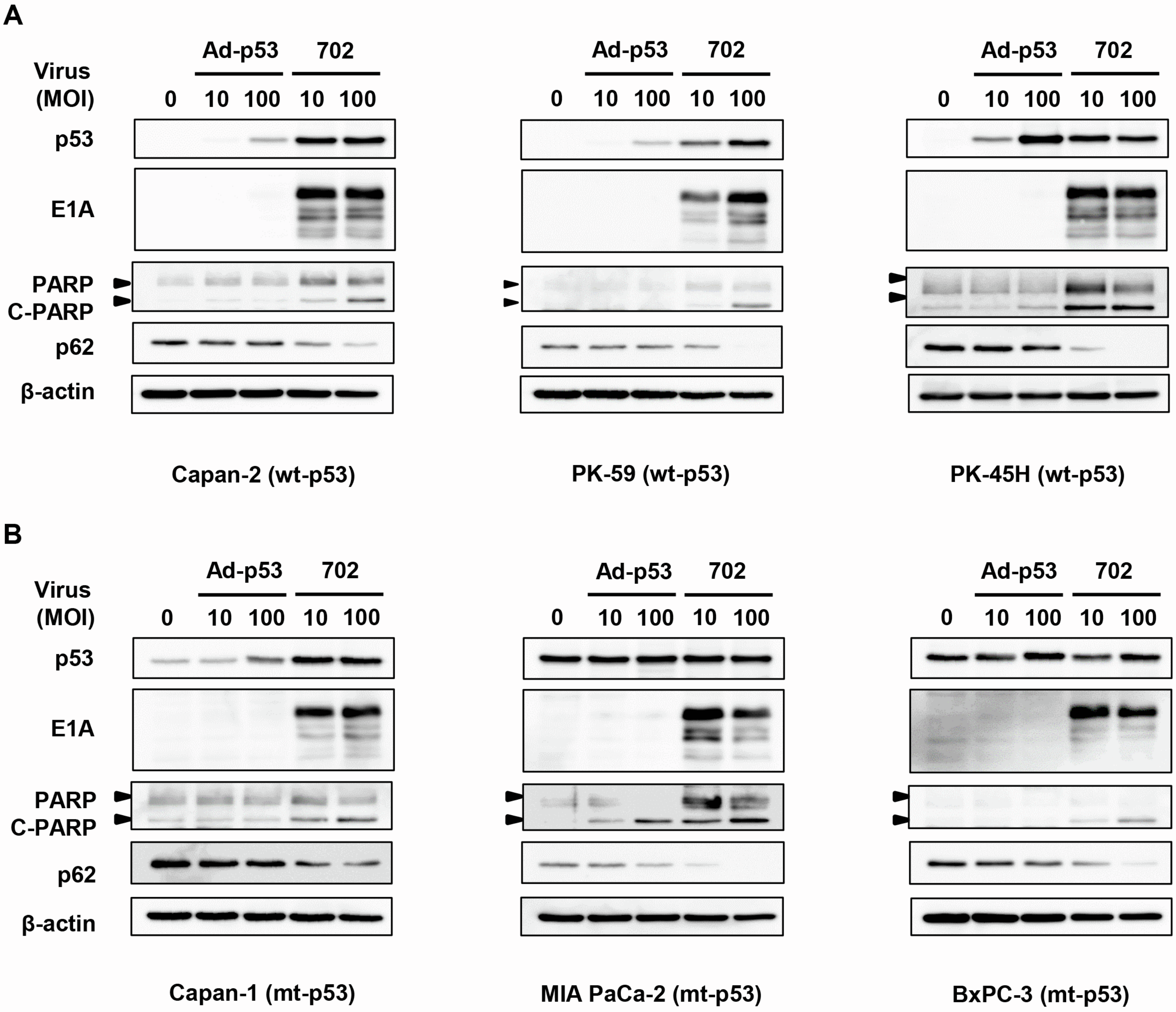

Figure S10. Therapeutic potential of Ad-p53 and OBP-702 to induce p53-mediated apoptosis and autophagy in human PDAC cells.

A, **B**, Human PDAC cells with wild-type p53 (wt-p53) (**A**) and mutant p53 (mt-p53) (**B**) were treated with Ad-p53 or OBP-702 at the indicated MOI for 48 h. Cell lysates were prepared and subjected to Western blot analysis of p53, E1A, PARP, cleaved PARP (C-PARP), and p62 expression. Β-actin was assayed as a loading control. Uninfected (mock-infected) cells were shown as virus-infected cells at an MOI of 0.

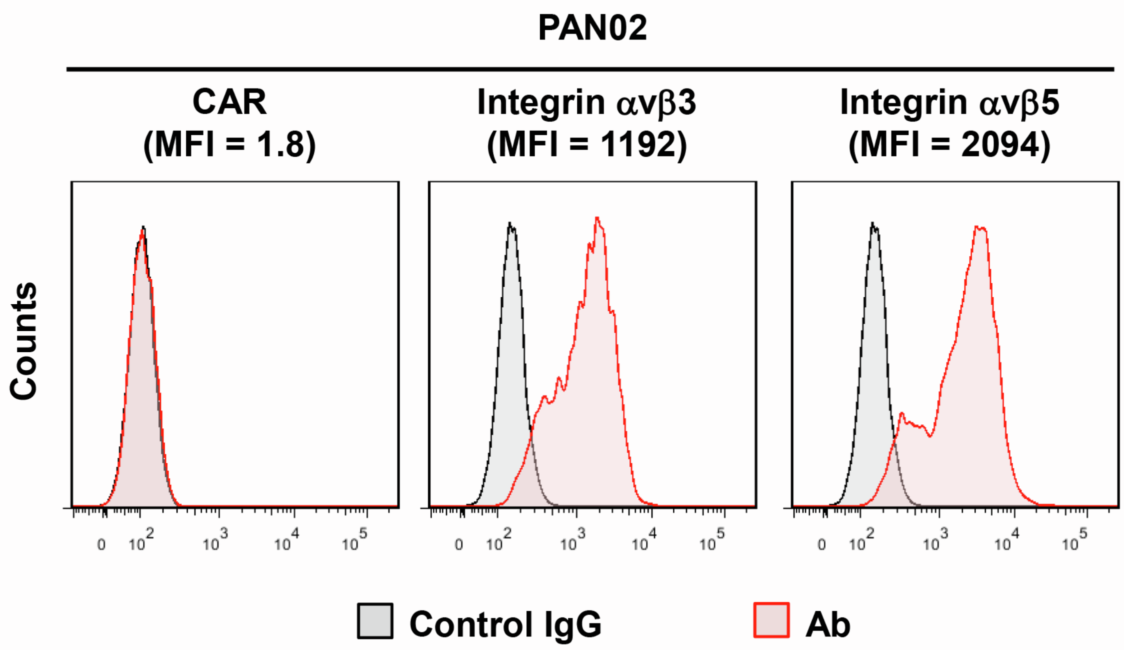

Figure S11. Expression of CAR and integrin proteins on the surface of murine PAN02 cells.

Expression levels of CAR, integrin $\alpha v \beta 3$, and integrin $\alpha v \beta 5$ on PAN02 cells were assessed by flow cytometric analysis. The mean fluorescence intensity (MFI) was determined by calculating the differences between the MFI of antibody (Ab)-treated and isotype control IgG-treated cells.

A

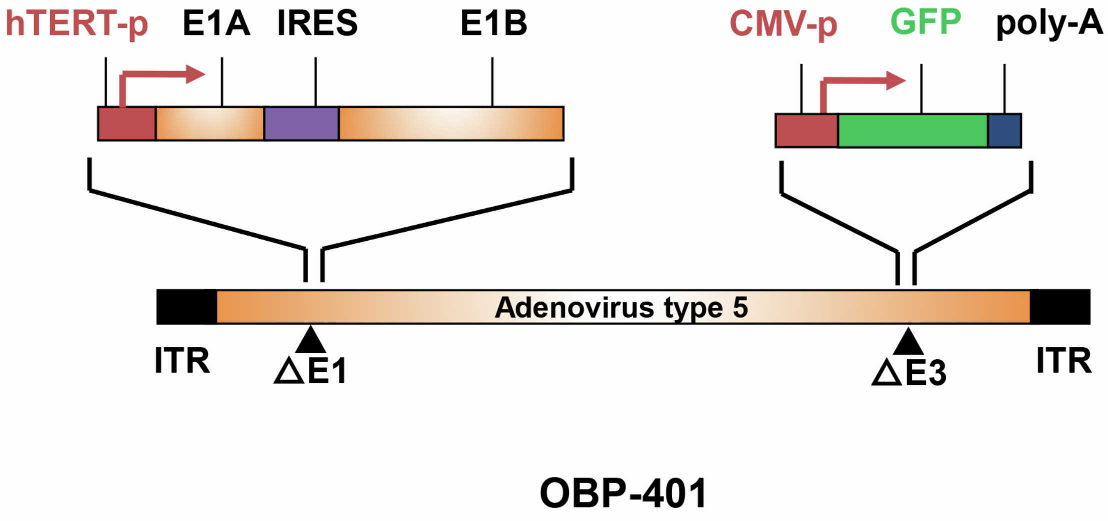

B

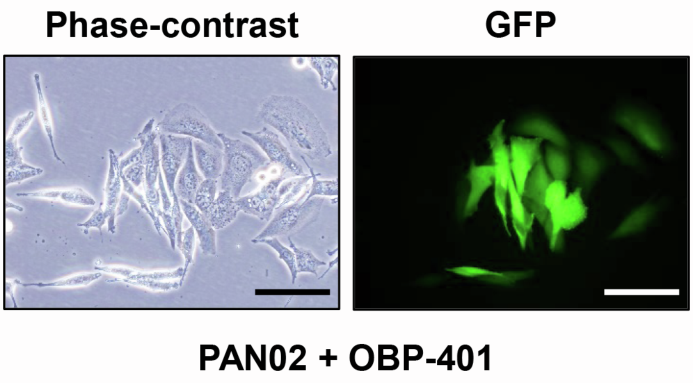

Figure S12. Induction of GFP expression in murine PAN02 cells by OBP-401 treatment.

A Genetic structure of OBP-401. **B** PAN02 cells were infected with OBP-401 at a MOI of 100 for 24 h. Expression of GFP was analyzed under fluorescence microscopy (IX71; Olympus). Scale bars, 100 μm.

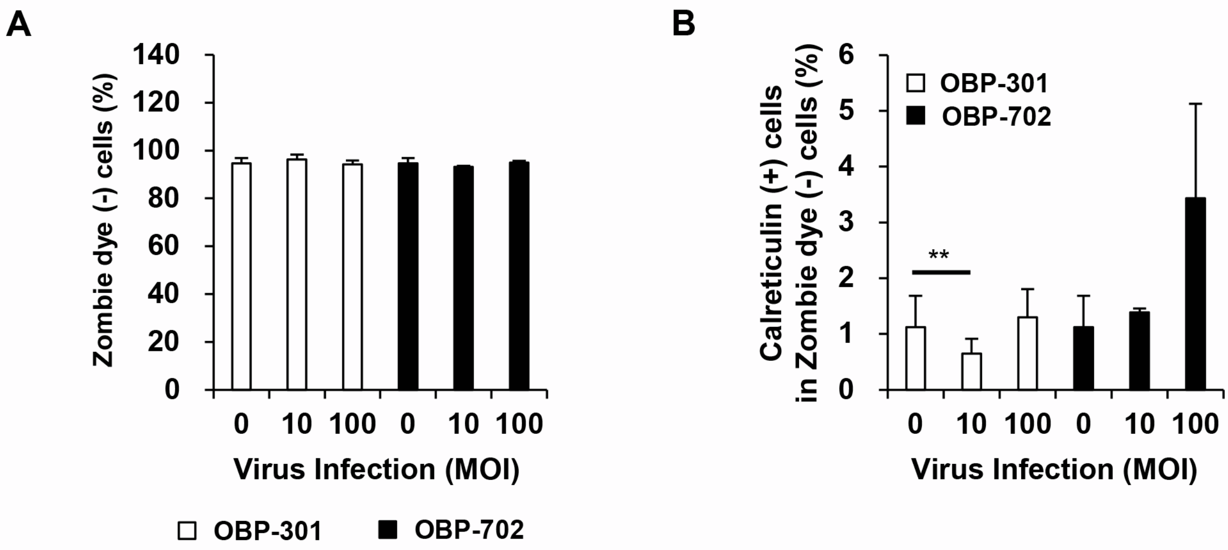

Figure S13. Proportion of zombie dye-unlabeled cells maintaining membrane integrity and cell surface calreticulin+ cells in murine PAN02 cells infected with Ad-p53 and OBP-702 for 48 h.

Murine PAN02 cells were treated with OBP-301 or OBP-702 at the indicated MOI for 48 h. **A**, The percentage of zombie dye-unlabeled cells maintaining membrane integrity was quantified by flow cytometry. **B**, The percentage of calreticulin+ cells among zombie dye-unlabeled cells maintaining membrane integrity was quantified by flow cytometry. Uninfected (mock-infected) cells were shown as virus-infected cells at an MOI of 0. Data are expressed as mean ± SD (n = 3). Student's *t* test was used to evaluate the significance of differences. **, $P < 0.01$ (versus an MOI of 0).

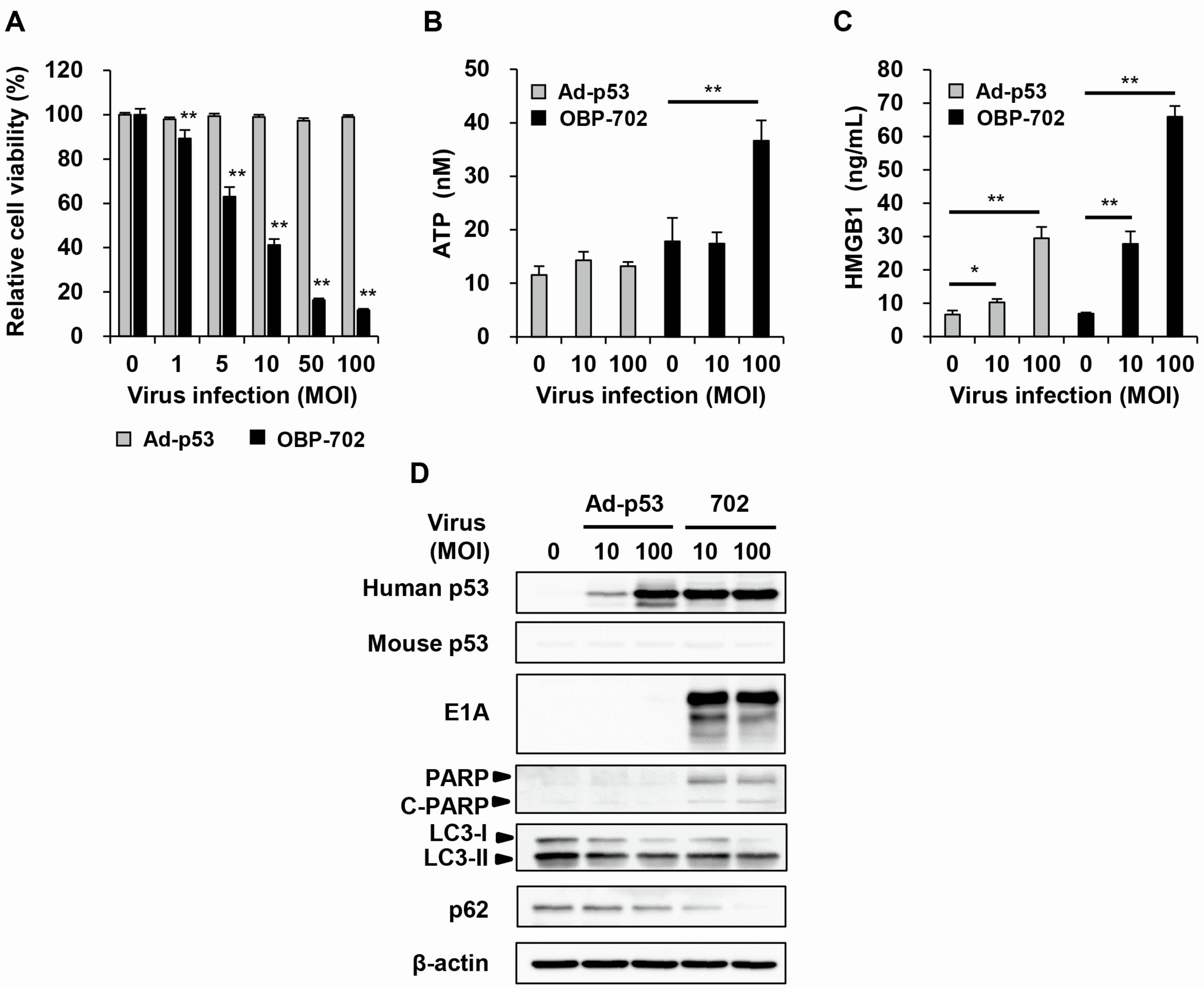

Figure S14. Therapeutic potential of Ad-p53 and OBP-702 to induce ICD in murine PAN02 cells.

Murine PAN02 cells were treated with Ad-p53 or OBP-702 at the indicated MOI for 48 h. Uninfected (mock-treated) cells were shown as virus-infected cells at an MOI of 0. A, Cell viability was quantified using the XTT assay. Data are expressed as mean ± SD (n = 5). **B**, **C**, Supernatants were collected, and the level of extracellular ATP (**B**) and HMGB1 (**C**) was determined using ENLITEN ATP assay and HMGB1 ELISA, respectively. Data are expressed as mean ± SD (n = 3). Student's *t* test was used to evaluate the significance of differences. *, $P < 0.05$; **, $P < 0.01$ (versus an MOI of 0). **D**, Cell lysates were prepared and subjected to Western blot analysis of human and mouse p53, E1A, PARP, cleaved PARP (C-PARP), LC3, and p62 expression. Β-Actin was assayed as a loading control.

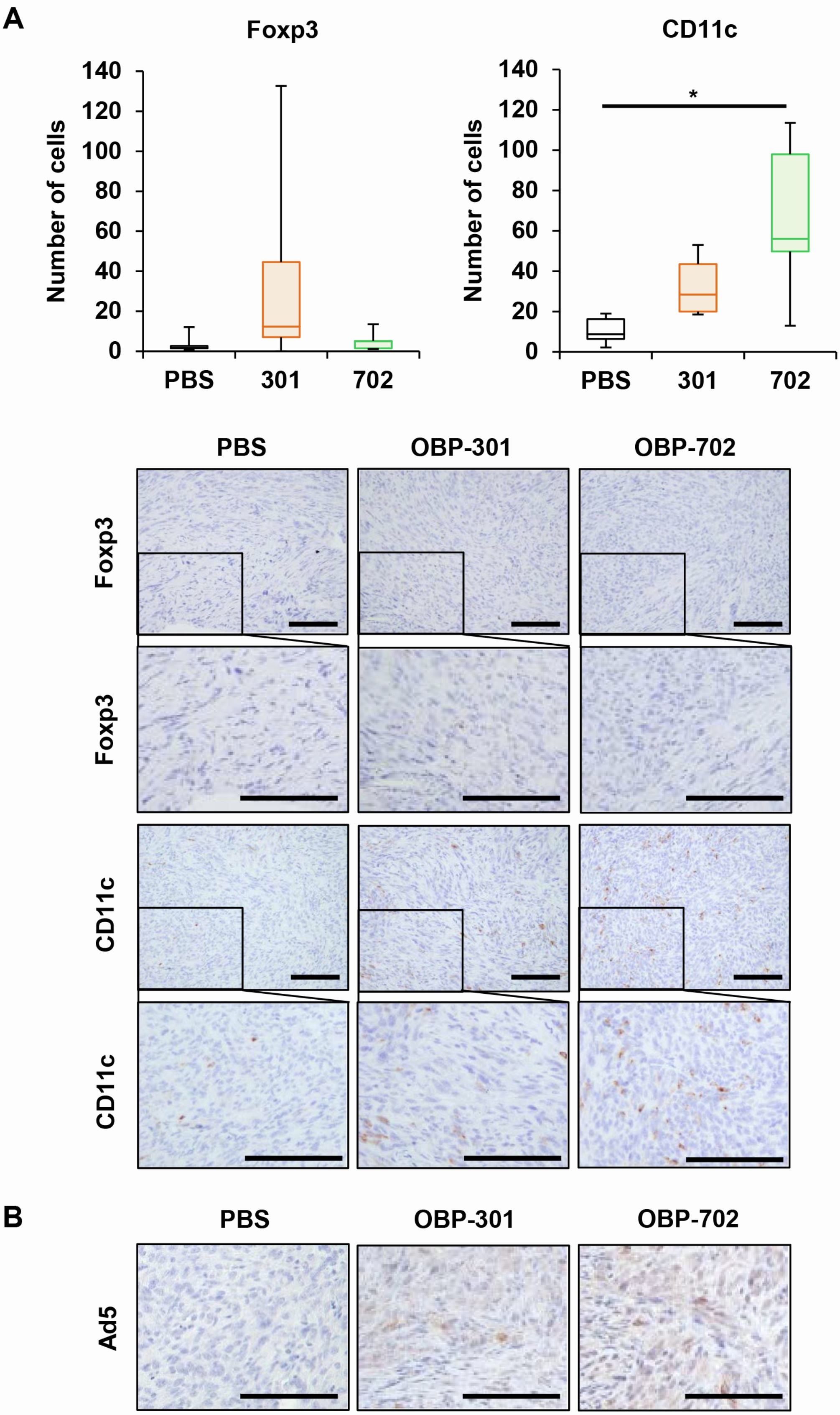

Figure S15. Number of Foxp3+ T cells and CD11c+ dendritic cells and adenovirus detection in murine PAN02 tumors treated with OBP-301 and OBP-702.

A The numbers of Foxp3+ T cells and CD11c+ dendritic cells were calculated from five randomly selected fields. Data are expressed as mean ± SD (n = 5). One-way ANOVA followed by the Games-Howell multiple comparison test was used to evaluate the significance of differences. *, $P < 0.05$. **B** Virus detection was performed by immunostaining using anti-Ad5 antibody. Scale bars, 100 μm.

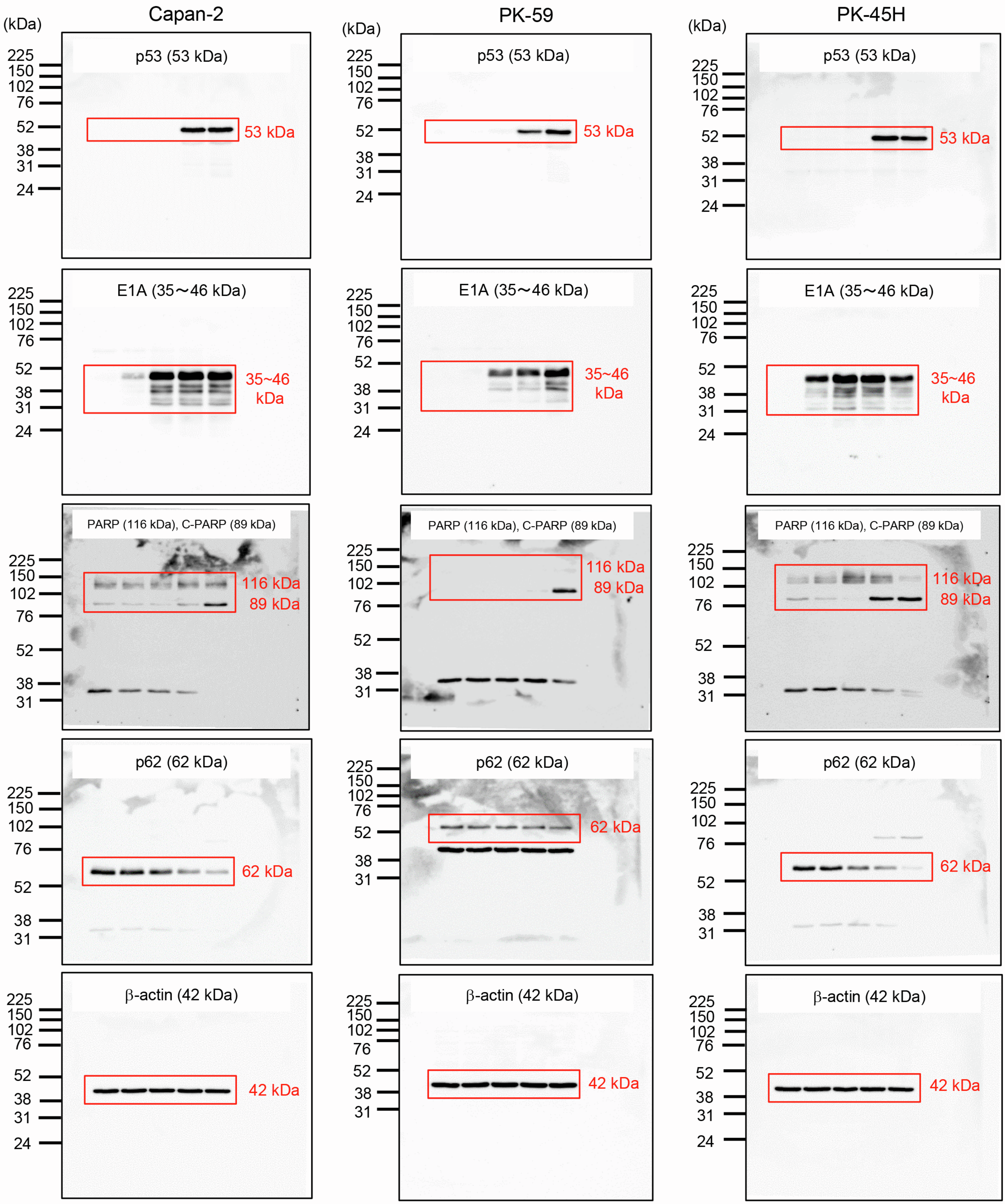

Figure S16. Full images of Figure 4A.

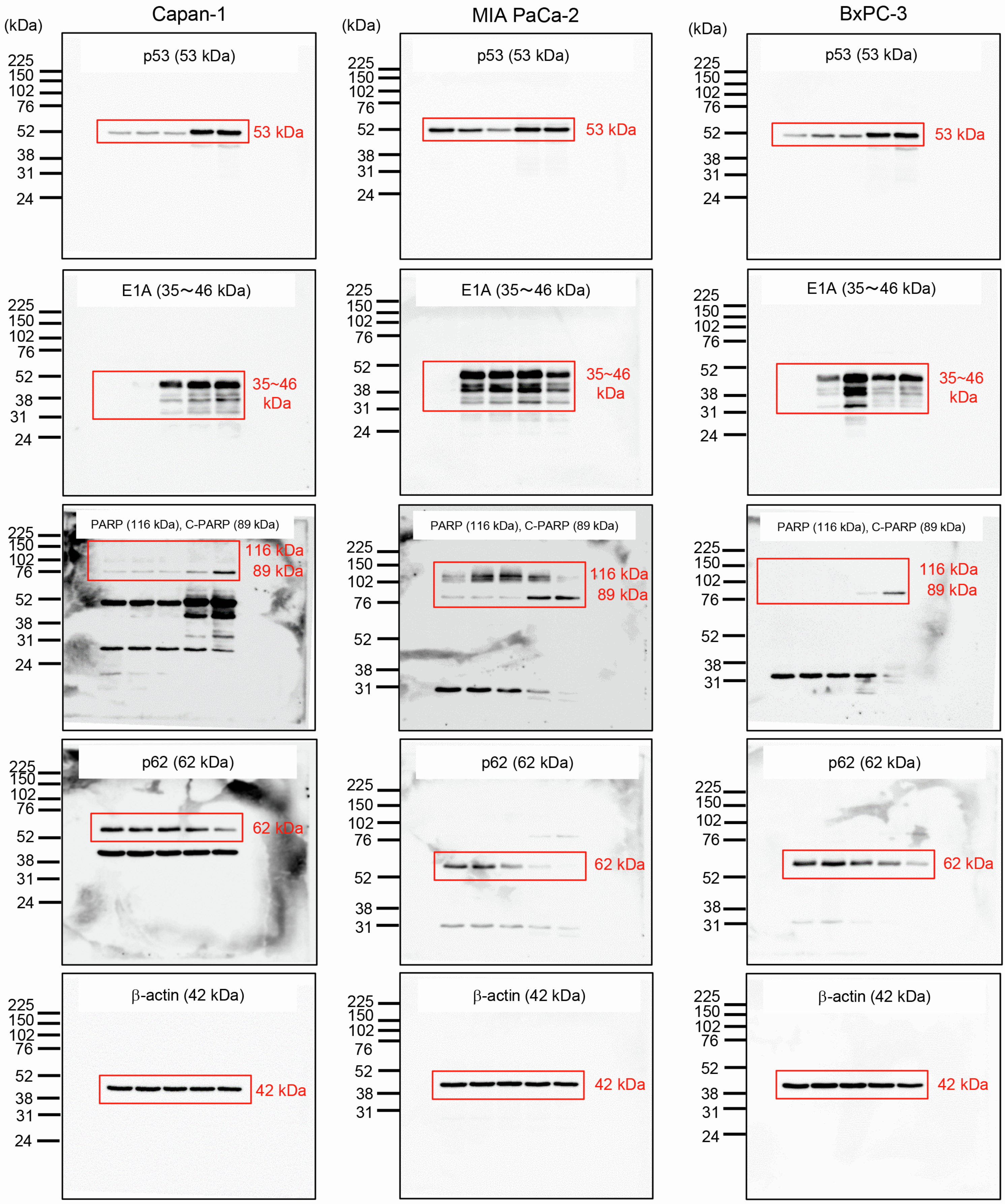

Figure S17. Full images of Figure 4B.

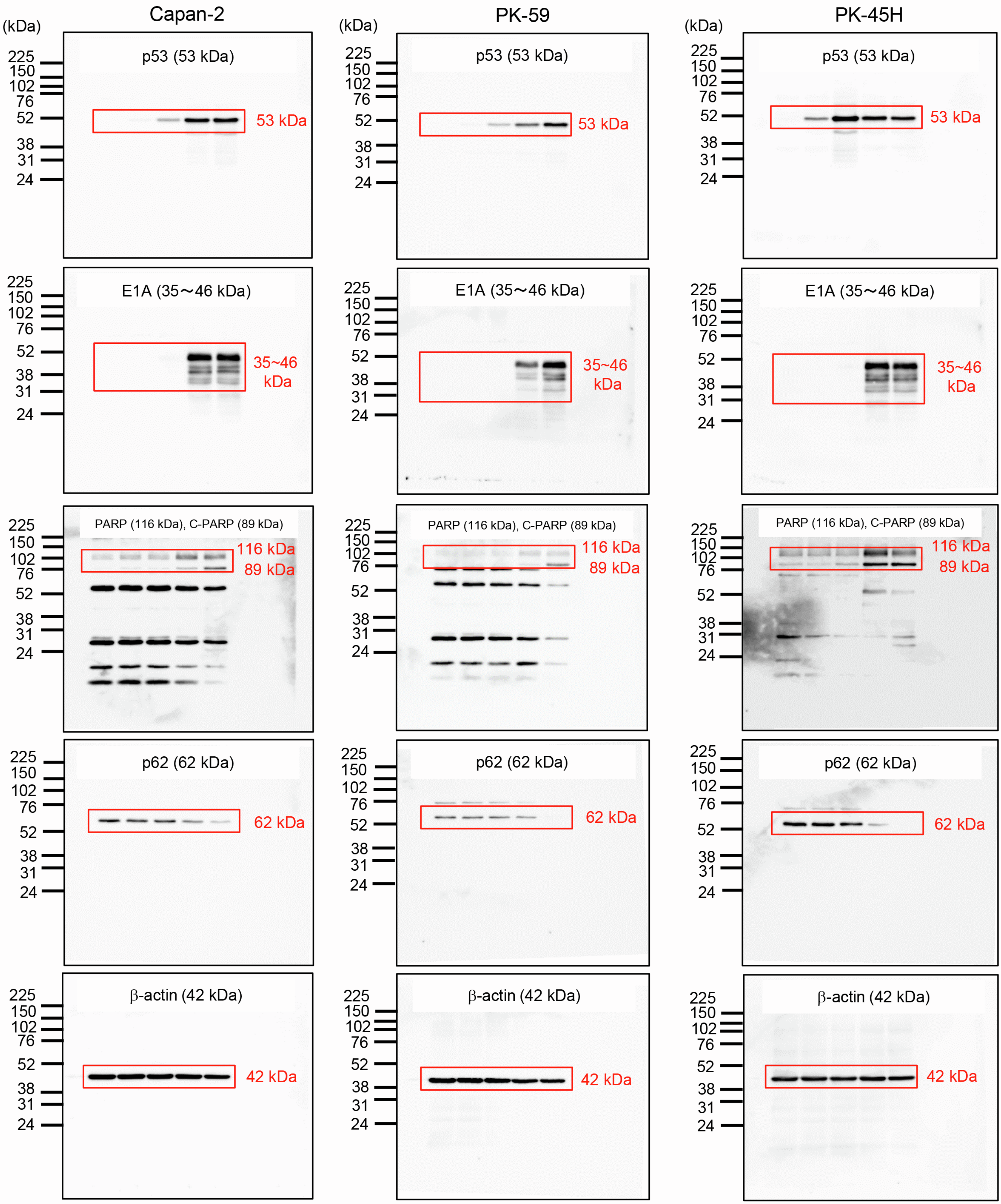

Figure S18. Full images of Figure S10A.

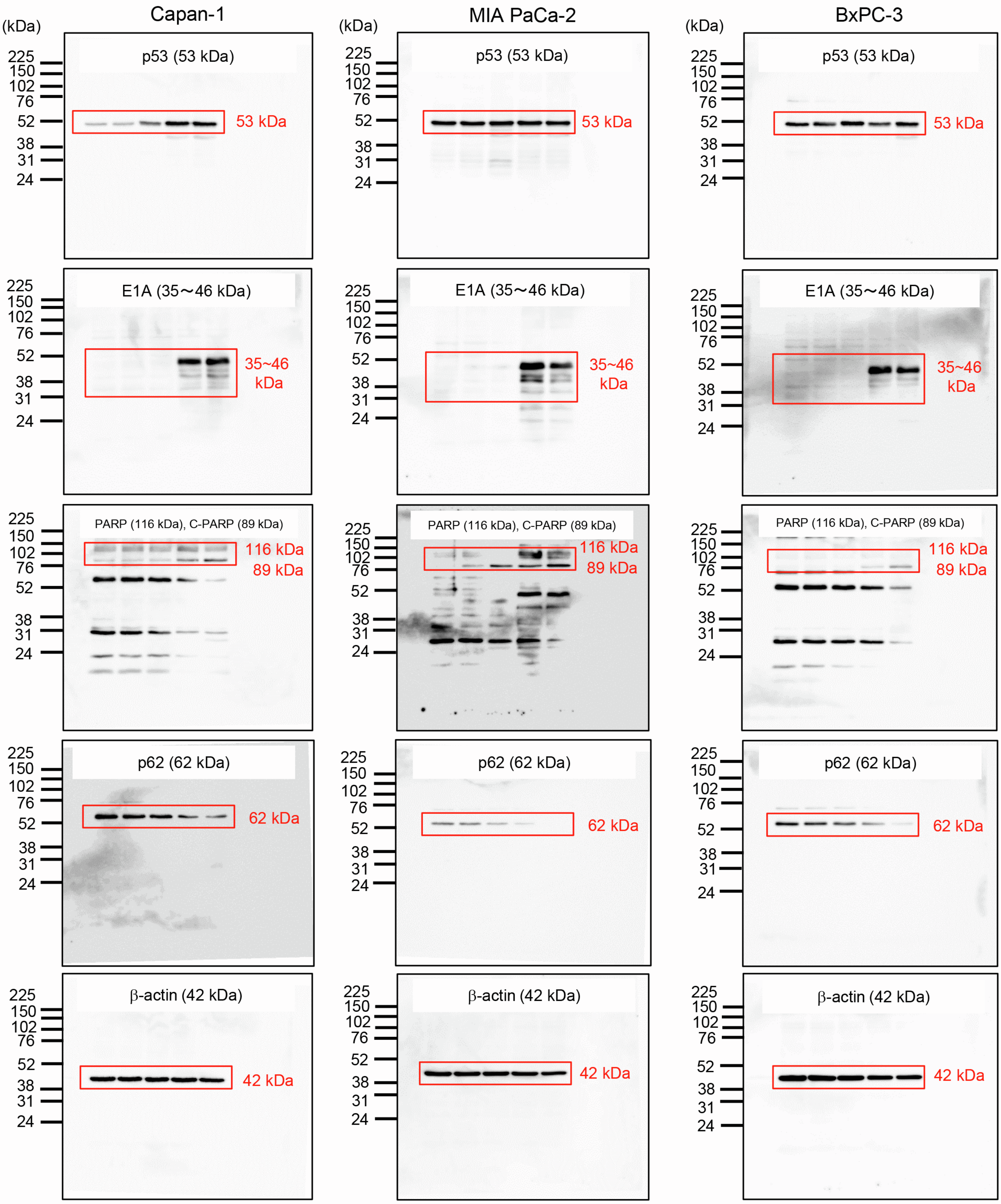

Figure S19. Full images of Figure S10B.

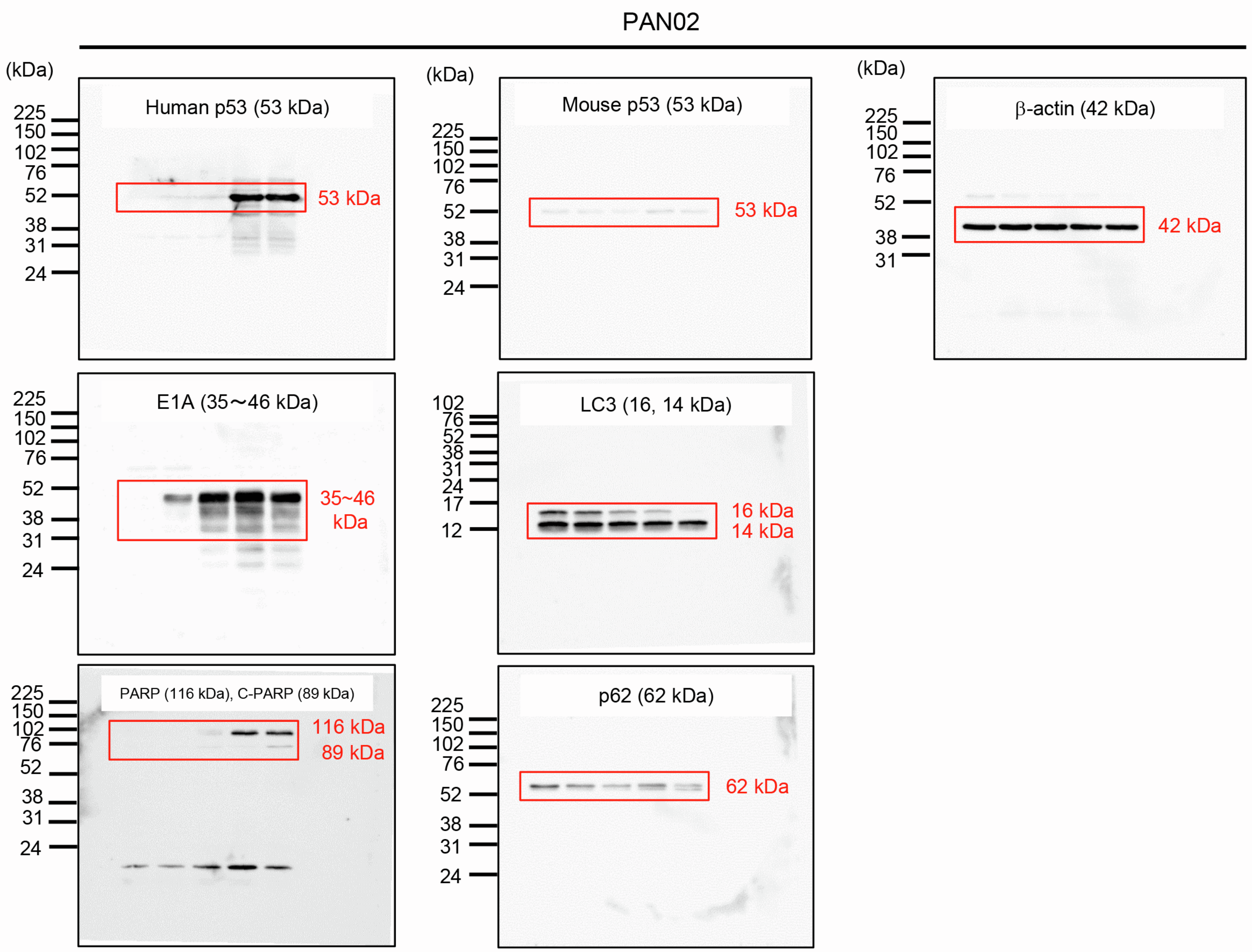

Figure S20. Full images of Figure 5D.

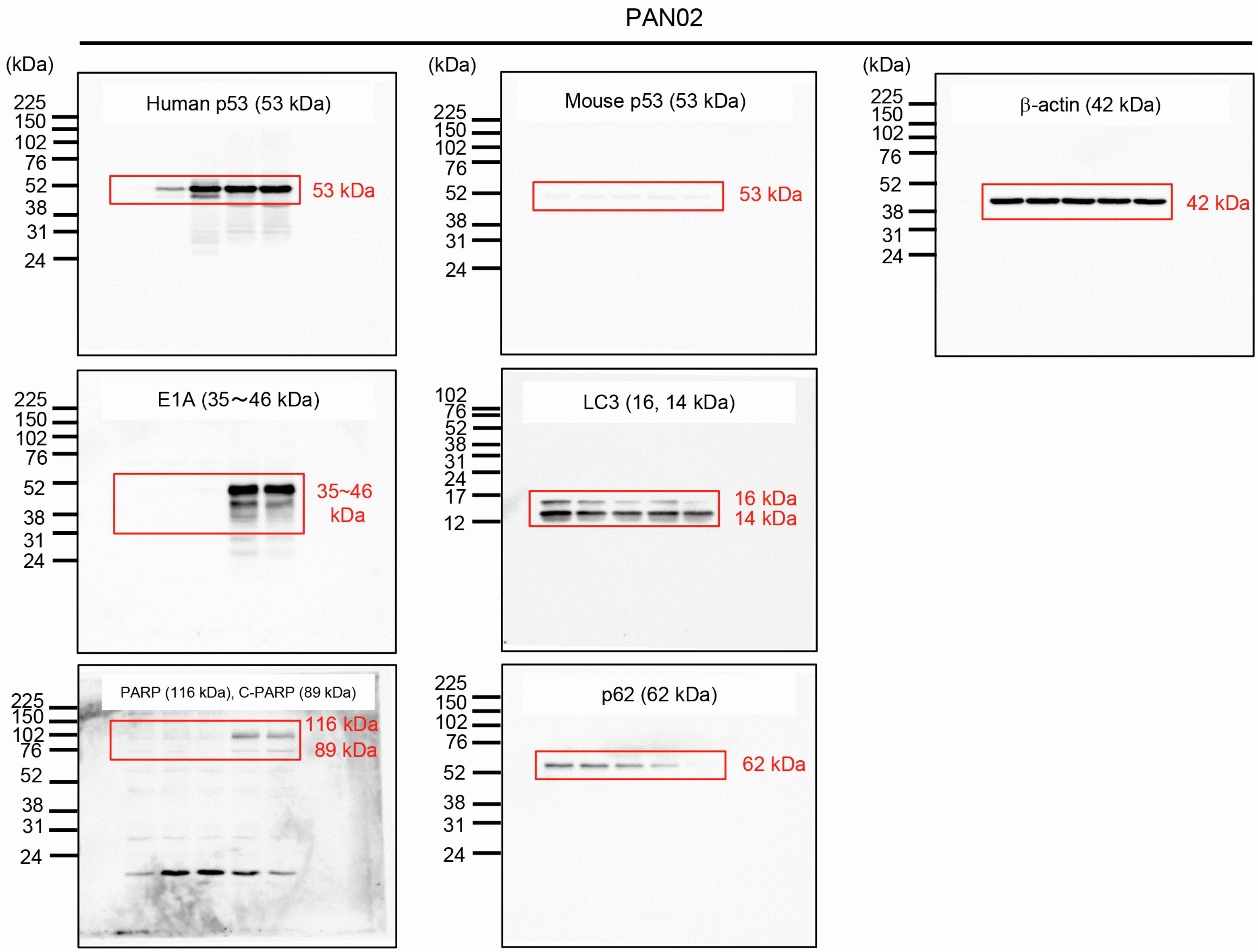

Figure S21. Full images of Figure S14D.
